# Single cell dynamics of tumor specificity vs bystander activity in CD8^+^ T cells define the diverse immune landscapes in colorectal cancer

**DOI:** 10.1038/s41421-023-00605-4

**Published:** 2023-11-15

**Authors:** Daniel Morales Borràs, Sara Verbandt, Markus Ausserhofer, Gregor Sturm, Jinyeong Lim, Gil Arasa Verge, Isaure Vanmeerbeek, Raquel S. Laureano, Jannes Govaerts, Jenny Sprooten, Yourae Hong, Rebecca Wall, Gert De Hertogh, Xavier Sagaert, Gabriele Bislenghi, André D’Hoore, Albert Wolthuis, Francesca Finotello, Woong-Yang Park, Stefan Naulaerts, Sabine Tejpar, Abhishek D. Garg

**Affiliations:** 1 Cell Stress and Immunity (CSI) Lab, Department of Cellular and Molecular Medicine, KU Leuven, Leuven, Belgium; 2 Digestive Oncology, Department of Oncology, KU Leuven, Leuven, Belgium; 3 Universität Innsbruck, Department of Molecular Biology, Digital Science Center (DiSC), Innsbruck, Austria; 4 Biocenter, Institute of Bioinformatics, Medical University of Innsbruck, Innsbruck, Austria; 5 Department of Health Sciences and Technology, Samsung Advanced Institute for Health Science and Technology, Sungkyunkwan University, Seoul, Republic of Korea; 6 Samsung Genome Institute, Samsung Medical Center, Sungkyunkwan University, Seoul, Republic of Korea; 7 Department of Pathology, University Hospitals Leuven, Leuven, Belgium; 8 Department of Abdominal Surgery, University Hospitals Leuven, Leuven, Belgium

**Keywords:** Tumour immunology, Tumour immunology, Bioinformatics, Cancer genetics, Colorectal cancer

## Abstract

CD8^+^ T cell activation via immune checkpoint blockade (ICB) is successful in microsatellite instable (MSI) colorectal cancer (CRC) patients. By comparison, the success of immunotherapy against microsatellite stable (MSS) CRC is limited. Little is known about the most critical features of CRC CD8^+^ T cells that together determine the diverse immune landscapes and contrasting ICB responses. Hence, we pursued a deep single cell mapping of CRC CD8^+^ T cells on transcriptomic and T cell receptor (TCR) repertoire levels in a diverse patient cohort, with additional surface proteome validation. This revealed that CRC CD8^+^ T cell dynamics are underscored by complex interactions between interferon-γ signaling, tumor reactivity, TCR repertoire, (predicted) TCR antigen-specificities, and environmental cues like gut microbiome or colon tissue-specific ‘self-like’ features. MSI CRC CD8^+^ T cells showed tumor-specific activation reminiscent of canonical ‘T cell hot’ tumors, whereas the MSS CRC CD8^+^ T cells exhibited tumor unspecific or bystander-like features. This was accompanied by inflammation reminiscent of ‘pseudo-T cell hot’ tumors. Consequently, MSI and MSS CRC CD8^+^ T cells showed overlapping phenotypic features that differed dramatically in their TCR antigen-specificities. Given their high discriminating potential for CD8^+^ T cell features/specificities, we used the single cell tumor-reactive signaling modules in CD8^+^ T cells to build a bulk tumor transcriptome classification for CRC patients. This “Immune Subtype Classification” (ISC) successfully distinguished various tumoral immune landscapes that showed prognostic value and predicted immunotherapy responses in CRC patients. Thus, we deliver a unique map of CRC CD8^+^ T cells that drives a novel tumor immune landscape classification, with relevance for immunotherapy decision-making.

## Introduction

Colorectal cancer (CRC) has two immunologically distinct tumor subtypes with microsatellite instability (MSI) phenotype associated with high mutational burden and high T cell infiltrates, vs tumors with microsatellite stability (MSS) exhibiting lower mutational burden and low T cell infiltrates^1–3^. Thus, these subtypes also exhibit distinct responses to immune checkpoint blockade (ICB)^3,4^.

ICBs typically induce tumor regression by ‘reinvigorating’ effector/cytotoxic functions of tumor-reactive CD8^+^ T cells^5–8^. This can overcome CD8^+^ T cell exhaustion induced by antigens and the microenvironment^5,6,8^. MSI CRC enriches for neoantigen-reactive CD8^+^ T cells and hence proficiently responds to programmed cell death 1 (PD-1) blockade^3,9^. However, MSS CRC has drastically lower neoantigen-enrichment thereby causing failure of PD-1 blockade^3^. Surprisingly, some MSS CRC patients can respond to PD-1 and cytotoxic T-lymphocyte associated protein 4 (CTLA4) co-blockade^10^. But this unexpected immuno-susceptibility is not sufficiently explained by the well-known low antigenic or ‘cold’ tumor labels applied to most MSS CRC tumors^2,4,11^. This exposes a major gap in our understanding of the clinico-immunological diversity underlying MSS CRC.

Transcriptomic analyses have classified CRC into various subtypes with immunological implications, e.g., consensus molecular subtype (CMS) or its refinements^12–14^ and various related frameworks capturing quantitative measurements for T cells in MSI/MSS CRC^1–3,15,16^. Latter frameworks often culminate into MSI-like MSS CD8^+^ T cell features. While these approaches have captured some prognostic impact of T cell infiltrates, they are yet to reliably predict the responses of specific MSS CRC patients to multimodal ICB regimens^3,10^. Also, their performance has been lackluster when differentiating durable responders to PD-1 blockade from non-responders in MSI CRC^3,9,17^.

To overcome these limitations, a biologically deep single cell dissection of CRC CD8^+^ T cells is required. Such dissection needs to be spearheaded by a systematic characterization of CD8^+^ T cell states and TCR repertoire between and within MSI and MSS CRC, to account for clinico-immunological heterogeneity. Recently, various pan-tumor single cell studies have highlighted the heterogeneity of tumor-reactive CRC CD8^+^ T cells^12,18–21^. However, many of these studies have either not deeply compared MSI to MSS CD8^+^ T cell profiles or have only revealed subtle differences in CD8^+^ T cell features that do not properly align with vast clinical and immunotherapeutic differences between MSI and MSS CRC. Thus, there is an urgent need to uncover differentiating features of CD8^+^ T cell states that properly align with clinically relevant immune diversity in CRC patients.

**Fig. 1:**
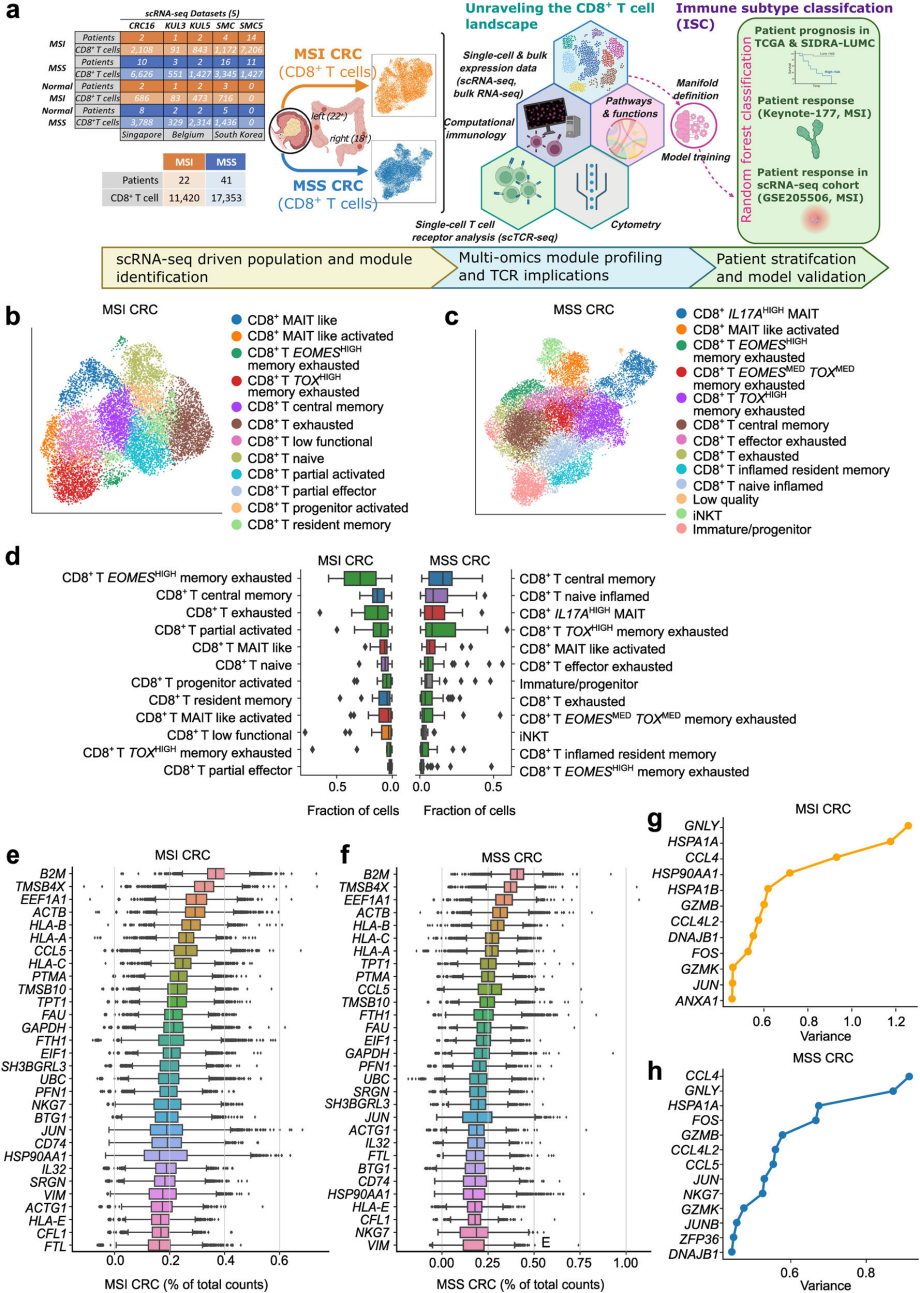
Multi-omics characterization of CD8^+^ T cell landscape for immune subtype classification. **a** Schematic overview of our computational CD8^+^ T workflow to differentiate tumor immune-induced responsiveness in CRC patients. 5’ 10X scRNA-seq datasets from Singapore, Belgium, and South Korea, spanning 11,420 CD8^+^ T cells across 22 MSI CRC patients and 17,353 CD8^+^ T cells across 41 MSS CRC patients. scRNA-seq profiles for CRC16 cohort, SMC, KUL3 and KUL5 (16,163 cells after quality filtering and doublet removal) have been previously published^12,18^ (4214 MSI, 11,949 MSS). Newly obtained data for this study include transcriptomes for 8,633 CD8^+^ T cells (7206 MSI, 1427 MSS) from the SMC5 cohort. Moreover, new single-cell TCR sequencing for the KUL5, SMC and SMC5 cohorts was added, covering 10,369 cells (5455 MSI, 4714 MSS) in 28 patients (16 MSI, 12 MSS). scRNA-seq expression modules were identified and subsequently translated to the TCGA bulk RNA-seq (374 patients retained with primary tumors, 57 MSI, 317 MSS) to obtain a novel CD8^+^ T cell-centric classification of CRC patients. This novel immune subtype classification (ISC) was validated for MSI and MSS CRC with regards to prognosis (TCGA, SIDRA-LUMC). Validations on ICB predictiveness were limited due to available data. MSI was verified in Keynote-177 (27 patients)^17^ and a public single cell dataset with pre- and post-ICB patient gene expression. The MSS situation was briefly assessed using public data from Parikh et al., which contains 8 pre-therapy CRC samples in a total of 24 CRC samples^103^. **b**, **c** UMAP representations of the CD8^+^ T cell population in MSI (**b**) and MSS (**c**). Groups were annotated after Leiden clustering post initial pre-processing control (filtering of low quality and doublet cells using Scanpy single cell analysis modules). **d** Relative abundance of CD8^+^ T cell populations per patient, sorted by median for both MSI and MSS CRC. **e**–**h** Top 30 highly expressed genes for MSI (**e**) and MSS (**f**) CRC as well as the top 13 variable genes for MSI (**g**) and MSS (**h**) CRC, sorted by gene variance, which highlight the elevated level of overlap of similar high expressed genes and variable genes between the MSI and MSS datasets.

To address this unmet need, we pursued a deep analysis of CRC CD8^+^ T cells on the levels of transcriptome (single cell RNA-sequencing (scRNA-seq)) and TCR repertoire (single cell TCR-sequencing (scTCR-seq)) followed by validation with proteomic mass cytometry by time-of-flight (CyTOF). Herein, single cell transcriptomes of 28,773 CD8^+^ T cells from 60 patients (11,420 CD8^+^ T cells from 22 MSI CRC patients and 17,353 CD8^+^ T cells from 41 MSS CRC patients) across five cohorts from Korea, Belgium, and Singapore, were integrated to create one of the largest CD8^+^ T cell single cell datasets with balanced coverage for MSI/MSS CRC (Fig. 1a). High quality TCR profiling (10X VDJ sequencing) was procured for 10,169 tumoral CD8^+^ T cells. Established CD8^+^ T cell features were interrogated on the levels of subpopulation phenotypes, interferon (IFN)-γ signaling, tumor-reactive signaling modules, TCR vs bystander activation, and TCR repertoire dynamics or epitope specificities (Fig. 1a). This revealed that MSI and MSS CRC had highly distinct tumor-reactive signaling and TCR specificities within the *IFNG*^+^CD8^+^ T cell compartment. Herein, we cross-connected the CD8^+^ T cell transcriptomic features with their predicted TCR antigen specificities, thereby revealing distinct tumor-reactive vs tumor irrelevant characteristics of CD8^+^ T cells infiltrating the MSI and MSS CRC tumors, respectively. Importantly, we also used the MSI/MSS-specific tumor-reactive signaling modules at the bulk tumor transcriptome level to create a manifold space, in which we aggregated MSI and MSS CRC patients in distinct subtypes with agglomerative clustering, using The Cancer Genome Atlas (TCGA) CRC dataset (Fig. 1a). This resulted in a novel immune classification system with seven subtypes, which could be predicted with ensemble machine learning. Our subtype classification framework identified two distinct MSI CRC patient groups (driven by stem-like signaling module) and two major MSS CRC patient groups (driven by inflamed memory-like module), which could be further divided in five heterogenous subgroups. The prognostic and immunotherapy response-predictive impact of this novel classification was also examined on independent clinical cohorts (Fig. 1a). Altogether, we highlight the ability of single cell CD8^+^ T cell-features to predict diverse tumor immune landscapes and differential immunotherapy-responses in CRC.

## Results

### A comprehensive map of CD8^+^ T cell subpopulations in MSI and MSS CRC

The single cell profiles for CD8^+^ T cells were extracted from scRNA-seq data of tumor tissue (28,773 CD8^+^ T cells) or normal-adjacent colon tissue (9825 CD8^+^ T cells) from five patient cohorts (Fig. 1a). These cohorts consisted of both newly generated (SMC5) and publicly available (CRC16, SMC, KUL3, KUL5) datasets (Fig. 1a). Samples were profiled via droplet-based scRNA-seq (10x technology) (see Supplementary Table S1 for patient details). These expression profiles were integrated and batch-corrected using Combat algorithm (Fig. 1a), and visual assessments revealed that the cells were sufficiently harmonized per cohort/dataset-level (Supplementary Fig. S1a, b). Of note, the performance of Combat was either similar to other popular batch correction methods (BBKNN, Harmony, Scanorama) or more stable than some (MNN correct, Regress Out) (Supplementary Fig. S1c, d). Importantly, unlike other studies, we batch corrected MSI or MSS CRC CD8^+^ T cells independently rather than jointly because we observed that while joint or independent batch correction didn’t affect the expression profile of MSS CRC CD8^+^ T cells yet joint batch correction significantly shifted the profile of MSI CRC CD8^+^ T cells closer to MSS CRC CD8^+^ T cells thereby rendering these cells more MSS-like in an artificial manner (Supplementary Fig. S1e). Hence, to uphold meaningful biological variation over mathematical imputations^22^ we continued with independent batch correction. In addition, cells were filtered to retain only high-quality cells and exclude doublets (see Materials and methods).

Next, subclusters of CD8^+^ T cells were identified using Leiden clustering, which resulted in 12 subclusters for MSI CRC CD8^+^ T cells (Fig. 1b) and 13 subclusters for MSS CRC CD8^+^ T cells (Fig. 1c). A comprehensive CD8^+^ T cell subpopulation annotation was performed based on well-known markers^6,8,23–25^ (Supplementary Table S2 and Fig. S2a–d). Annotations of the conventional CD8^+^ T cells used established markers of naive/progenitor, memory, exhaustion, or effector/activation profiles (Supplementary Table S2 and Fig. S2a–d). Some ‘unconventional or invariant’ subpopulation annotations were based on *CD8A/B* expression (Supplementary Fig. S2a, b), including cells with *CD8A*^+^*CD8B*^LOW^ (thus, *CD8A* > *CD8B*) profiles that were annotated as CD8^+^ mucosal associated invariant T cells (MAIT), as compared to *CD8A*^HIGH^*CD8B*^+/MED/HIGH^ profiles of the other conventional CD8^+^ T cells^26,27^ (Supplementary Fig. S2a, b). Indeed, we cross-validated that more cells in the MAIT-annotated or MAIT-like groups highly expressed a MAIT-specific genetic signature than those in the conventional CD8^+^ T cell cluster^28,29^ (Supplementary Fig. S2e and Table S3). Cells with *CD8A*^LOW^*CD8B*^LOW^ profiles were annotated as immature/progenitor (*IL7R*^HIGH^ ^25^) or invariant natural killer-T (iNKT) cells (*IL2RB*^HIGH^)^30^ (Supplementary Fig. S2c, d). Finally, cells with negligible expression of *CD8A/B* were annotated as low quality (Supplementary Fig. S2d). Altogether, we assembled a comprehensive annotation of CRC-CD8^+^ T cells that spanned across tumor subtype pathology (MSI vs MSS), patient geographical origin (3 countries across two continents) and dataset heterogeneity (5 cohorts).

### Subpopulation phenotypes of CD8^+^ T cells overlap between MSI and MSS CRC

When comparing the CD8^+^ T cell subpopulation phenotypes between MSI and MSS CRC, the top 5 most enriched subpopulations in MSI CRC were *EOMES*^*HIGH*^CD8^+^ T memory-exhausted, CD8^+^ T central-memory, CD8^+^ T exhausted, CD8^+^ T partially activated and MAIT-like (Fig. 1d), aligning with the established tendency of MSI CRC to enrich memory/exhaustion signaling^1,18^. The top 5 most enriched subpopulations in MSS CRC were CD8^+^ T central-memory, CD8^+^ T naive-inflamed, *IL17A*^*HIGH*^ MAITs, *TOX*^*HIGH*^*CD8*^+^ T memory-exhausted and MAIT-like activated (Fig. 1d), aligning with the known mixed inflammatory phenotypes of MSS CRC^1,18^. Nevertheless, both MSI and MSS CRC CD8^+^ T cell subpopulations showed variable overlaps for the following states: effector/activated (*IFNG, TNF, PRF1, CCL5* and/or *GZMA/B/K*), memory-exhausted (*TOX*, *EOMES*), or exhaustion (immune-inhibitory receptor (IR) genes: *HAVCR2, ENTPD1, CTLA4, LAG3, TIGIT*, and/or *PDCD1*) (Supplementary Table S2 and Fig. S2c, d). Since such effector/activated, memory-exhausted, exhaustion states are surrogate markers of tumor reactivity in ICB responsive tumors, like melanoma or lung cancer^8,25,31^, the above overlaps between MSI and MSS CRC subpopulation phenotypes were unexpected^1,32^. In addition, MSS CRC enriched for more *EOMES*^*MED*/HIGH^CD8^+^ T cells than MSI CRC (Supplementary Fig. S2c, d). While this can be a sign of tumor reactivity^33^, it can also imply long-lived memory cells specific for tumor irrelevant antigens (e.g., self/viral antigens) or bystander memory phenotypes^34,35^. Interestingly, some unconventional/invariant CD8^+^ T cell subpopulations were mainly present in MSS CRC (i.e., *IL17A*^+^ MAITs, iNKT cells, or immature cells) (Fig. 1d; Supplementary Fig. S2a, b).

Next, we wondered if these overlaps also existed for the top expressed or most variable genetic modules. Across CD8^+^ T cells, in both MSI and MSS CRC, there were similar highly expressed genes, relevant for antigen-presentation or activation (*B2M, HLA-A/B/C/E, CD74*), effector activity (*CCL5, NKG7, SRGN*) or inflammation (*JUN, IL32*) (Fig. 1e, f). Even for the most variable genes across CD8^+^ T cells, there was considerable overlap between these CRC subtypes for genes relevant for cytolytic activity (*GNLY, GZMB, GZMK*) or inflammation (*CCL4, CCL4L2, FOS, JUN*) (Fig. 1g, h). Thus, despite well-established antigenic distinctions between MSI and MSS CRC and their independent batch correction to uphold biological variation, there was a substantial overlap in conventional subpopulation phenotypes and broad expression modules of their CD8^+^ T cells.

### *IFNG*^+^CD8^+^ T cells and per cell *IFNG* expression show contrasting patterns in CRC subtypes

In the above transcriptomic overlaps, IFN-γ signaling was missing, despite its critical role in effector function, including synergizing with TCR stimulation to sustain IR expression and exhaustion^36^. Moreover, some biomarkers centered on IFN-γ^+^ T cells are amongst the most consistent predictors of ICB-responses in multiple cancers^37^. Therefore, we pursued a deep dissection of the IFN-γ signaling.

**Fig. 2:**
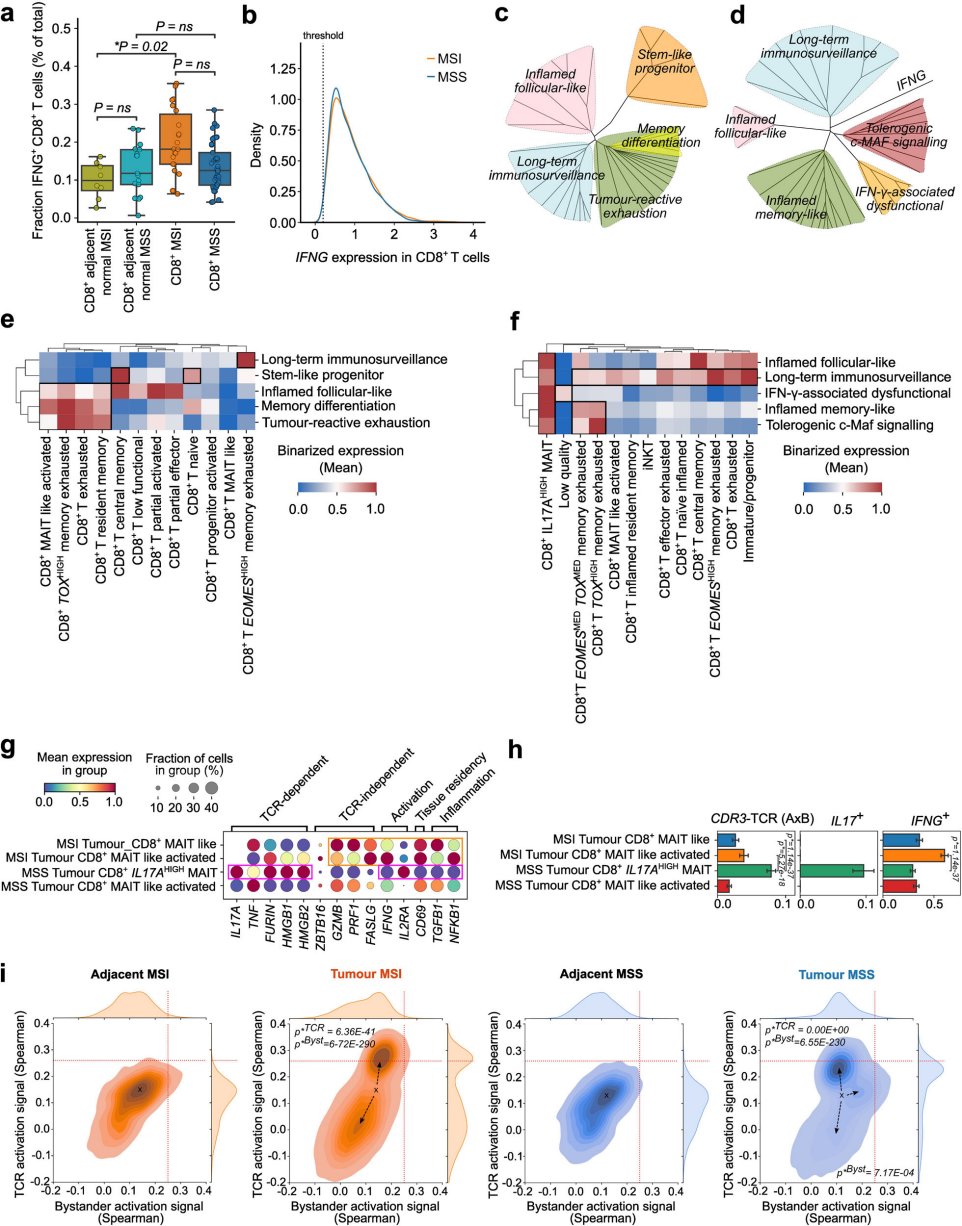
*IFNG*^+^CD8^+^ T cells, IFNG expression and tumor-reactive signaling. **a** Proportion of total CD8^+^ T cells positive for *IFNG* expression per patient. 1958 CD8^+^ T cells in normal tissue adjacent to MSI (green), 7867 CD8^+^ T cells in normal tissue adjacent to MSS CRC (light blue), 11,420 tumoral MSI CD8^+^ T cells (orange) and 17,353 tumoral CD8^+^ T cells (blue) were analyzed. Median (line) quartile 2 and 3 (box), as well as quartiles 1 and 4 (whiskers) are indicated. Pairwise contrasts were calculated with Welch’s two-sided *t*-test (*P* < 0.05). **b** Density plot indicating the distribution of CD8^+^ T cells over the expression level of *IFNG* in CD8^+^ T cells present in MSI (11,420 cells) and MSS (17,353 cells) tumors. **c**, **d** Genetic signaling of co-expression correlation matrices, visualized as unrooted hierarchical distance for MSI (**c**) and MSS (**d**), highlighting the different modules characterizing *IFNG*^+^CD8^+^ T cells. **e**, **f** Activation map (binarized expression) for tumor-reactive signaling modules (Supplementary Table S2) for each sub-population of *IFNG*^+^CD8^+^ T cells in MSI (**e**) and MSS (**f**). **g** Dot plot of *IFNG*^+^ MAIT cells in MSI and MSS. These cells are a source of TCR-dependent and independent, activation, tissue residency and inflammation genetic signal. **h** Bar charts of average local alignment identity of TCR alpha and beta chains as well as binarized count of *IL17*^+^ cells and binarized counts of *IFNG*^+^ cells (statistical significance calculated using two-sample independent *t*-test, *P* value threshold < 0.05). **i** Joint topographical density plot between TCR activation signaling (*y* axis) and bystander activation signaling (*x* axis) for *IFNG*^+^CD8^+^ T cells including normal MSI (orange left), tumor MSI (orange right), normal MSS (blue left), and tumor MSS (blue right) with arrows indicating the population drifts from normal tissue mean population center (*x*). Quadrants defined as vertical and horizontal lines were used to calculate Welch test statistical cell quantification differences between MSI and MSS tumor samples (*P* value threshold < 0.05).

We divided the total CD8^+^ T cells for both MSI and MSS CRC into *IFNG*^+^ and *IFNG*^*–*^subpopulations based on their transcript expression (threshold of 0.2 log_2_-normalized CPM). This threshold efficiently distinguished CD8^+^ T cells with not too low *IFNG* expression from CD8^+^ T cells with clear expression of *IFNG* (Supplementary Fig. S3a). We took along *IFNG*^+^CD8^+^ T cells from normal-adjacent colon tissue for peri-tumoral comparison. Expectedly, MSI CRC had the highest amounts of *IFNG*^+^CD8^+^ T cells compared to both normal-adjacent tissue and MSS CRC (Fig. 2a). MSS CRC also did not show a higher proportion of *IFNG*^+^CD8^+^ T cells than its normal-adjacent tissue (Fig. 2a). However, the total distribution of *IFNG* expression per CD8^+^ T cells was similar between MSI and MSS CRC (Fig. 2b). Accordingly, there was a very marginal difference in downstream IFN-γ response signature^38^ in these MSI/MSS CRC *IFNG*^+^CD8^+^ T cells (Supplementary Fig. S3b). Thus, the lower numbers of *IFNG*^+^CD8^+^ T cells in MSS CRC exhibited similar *IFNG* output as well as downstream IFN-γ signaling, as the somewhat higher numbers of *IFNG*^+^CD8^+^ T cells in MSI CRC.

### MSI and MSS CRC show contrasting tumor-reactive signaling in IFNG^+^CD8^+^ T cells

Increased IFN-γ signaling is a hallmark of heightened tumor reactivity^39^, and thus above similarities between MSI and MSS CRC were surprising. This highlighted a need for comprehensive analyses of various genes relevant for tumor-reactive signaling, including exhaustion, to understand the immunodynamics of IFN-γ signaling. Hence, we analyzed major genes relevant for tumor-reactive CD8^+^ T cell exhaustion (*TIGIT, PDCD1, LAG3, CTLA4, HAVCR2, ENTPD1, TCF7, TOX, EOMES, CXCL10, CXCL9, CXCL13, CXCR5, ICOS, IL18R1, IL15RA, IL18RAP, KLRG1, KLRK1, BCL6, SLAMF6*)^40^ (Supplementary Table S3), and critical cytokine or TCR signaling related components as well as transcription factors (TFs) upstream of exhaustion-relevant IR-coding genes (*IL2, CD28, TNF, SMAD3, SMAD4, SMAD2, NFATC1, NFATC3, NFATC2, MAF, STAT2, STAT1, JAK3, PRDM1, TBX21, YY1, NFIL3*)^36^. To validate the ability of these genes to mark tumor-reactive exhaustion, we performed an unbiased pathway overrepresentation analyses^41^. Indeed these genes together enriched for various pathways relevant for tumor-reactive exhaustion or multi-factorial tumoral inflammation^7,31,42,43^ including nuclear factor of activated T cells (NFAT) signaling, IFNG signaling, various IL-based pathways including IL2/IL15, TGFβ signaling, and various T cell-relevant transcriptional pathways (Supplementary Fig. S3c).

Quantitatively, these genes were mostly expressed in *IFNG*^+^CD8^+^ T cells in both MSI and MSS CRC (Supplementary Fig. S3d). For several key regulators coding for exhaustion-relevant IRs/TFs, the gene expressions between MSI and MSS CRC *IFNG*^+^CD8^+^ T cells were not different, e.g., *NFATC3, STAT2, PRDM1, STAT1, TIGIT, PDCD1, TBX21*, *TOX* (Supplementary Fig. S3d). However, other genes showed higher expression in MSI CRC, e.g., *LAG3, CTLA4, CXCL13, HAVCR2, ENTPD1, YY1, NFATC2, JAK3* (Supplementary Fig. S3d). This indicated a relatively higher tumor reactivity in MSI CD8^+^ T cells, as documented recently for *CXCL13*^*+*^CD8^+^ T cells in MSI CRC^1^.

This, however, did not clarify the tumor reactivity situation for MSS CRC. Hence, we pursued a qualitative analysis of the above genes, along with *IFNG*, i.e., the tendency of these genes to sub-cluster *IFNG*^+^CD8^+^ T cell populations in MSI vs MSS CRC. Using correlation matrices and hierarchical clustering with Spearman correlation, we were able to better identify contrasting patterns in MSI and MSS CRC. More elaborate discussion on how different modules based on same genes were differently labeled between MSI and MSS CRC was shown in Supplementary Table S4. MSI CRC *IFNG*^+^CD8^+^ T cells showed 5 clusters with distinct signaling modules: stem-like progenitor (distinguished by *TCF7, IL2, CD28*)^40^, inflamed follicular-like (*CXCR5, BCL6, ICOS*)^44^, long-term immunosurveillance (*EOMES, TBX21, PRDM1, KLRK1, KLRG1*)^45,46^, tumor-reactive exhaustion (*IFNG, LAG3, PDCD1, CTLA4, TIGIT, ENTPD1, HAVCR2, CXCL13, NFATC2*)^8,24,47^ and memory differentiation (*TOX, IL15RA, NFATC3, NFIL3, STAT1/2*)^36,40^ (Fig. 2c; Supplementary Fig. S3e and Table S3). The memory differentiation module was fully integrated within the broader tumor-reactive exhaustion module (Fig. 2c; Supplementary Fig. S3e). The stem-like progenitor module had the most distinct existence in MSI CRC (Fig. 2c; Supplementary Fig. S3e), in line with observations in melanoma^48^.

MSS CRC *IFNG*^+^CD8^+^ T cells showed 5 subclusters (Fig. 2d; Supplementary Fig. S3f): long-term immunosurveillance (*EOMES, PRDM1, KLRK1, KLRG1, IL2, TNF*)^45,46^, inflamed follicular-like (*CXCR5, BCL6, CD28*)^44^, inflamed memory-like (*TOX, PDCD1, CTLA4, NFATC2/3, SMAD2/3/4, STAT1/2, IL15RA, IL18RAP*), IFN-γ-associated dysfunctional (positive correlations with IFN-γ-related genes like *TBX21, YY1, NFIL3, CXCL9, CXCL10* but negative correlations with other functional genes like *IL2, TNF, KLRK1*)^49^ and tolerogenic c-Maf signaling (*MAF, ICOS, LAG3, TIGIT, ENTPD1, HAVCR2*)^50^ (Supplementary Table S3). Unlike the tumor-reactive exhaustion module in MSI CRC, none of the specific subclusters of *IFNG*^+^CD8^+^ T cells in MSS CRC recruited *IFNG* (Fig. 2d; Supplementary Fig. S3f), possibly indicating a lack of immunological orientation for *IFNG*^51^. In addition, the only two clusters that showed some overlap between MSS and MSI CRC (Fig. 2c, d; Supplementary Fig. S3e, f) also showed varied sizes in MSS CRC in terms of number of genes: long-term immunosurveillance was bigger and inflamed follicular-like was smaller. We did not identify a distinct tumor-reactive exhaustion cluster in MSS CRC. Instead, the IR-coding genes and *TOX*, co-markers of tumor-reactive exhaustion^52,53^ were split across two distinct clusters, inflamed memory-like and tolerogenic c-Maf signaling (Fig. 2d; Supplementary Fig. S3f). The fragmentation of exhaustion markers across subpopulations, rather than co-association, is considered a marker of ICB non-responsive tumors with bystander CD8^+^ T cells^34,54^. In addition, c-Maf signaling specifically distinguished MSS CRC CD8^+^ T cells (Fig. 2d; Supplementary Fig. S3f). This is of significance because c-Maf signaling in CD8^+^ T cells associates with tolerogenic/non-inflammatory functions, involved in tumor immune-escape^50^, which is particularly pertinent for colonic surfaces where c-Maf signaling facilitates tolerogenicity towards gut microbiome^50^. Of note, there was some but not extremely high genetic overlaps between the above 5 MSI and 5 MSS CRC signaling modules (Supplementary Fig. S3g). Altogether, we observed that MSI CRC *IFNG*^+^CD8^+^ T cells have tumor-reactive exhaustion and stem-like progenitor signaling, while MSS CRC *IFNG*^+^CD8^+^ T cells showed heterogeneous inflammatory or tolerogenic features.

### MSI and MSS CRC are composed of highly distinct CD8^+^ T cell signaling hubs

Next, we created an alignment map of subpopulation annotations of CD8^+^ T cells with the tumor-reactive signaling modules, to uncover putative CD8^+^ T cell signaling hubs^1^. This was meant to highlight how CD8^+^ T cell sub-populations diverge (negative or null correlation) or converge (positive correlation) with respect to signaling signatures thereby revealing cross-connected CD8^+^ T cell hubs.

In MSI CRC, tumor-reactive exhaustion and memory differentiation aligned with *TOX*^HIGH^CD8^+^ T memory-exhausted, CD8^+^ T exhausted and CD8^+^ T resident-memory cells (Fig. 2e), whereas the stem-like progenitor module aligned primarily with CD8^+^ T central-memory and CD8^+^ T naive cells (Fig. 2e). This was in line with stem-like memory cells’ known tendency to show both naive and central memory T cell markers^55,56^. Finally, the inflamed follicular-like module^57^ broadly aligned with multiple CD8^+^ T cell subpopulations while the long-term immunosurveillance only aligned with *EOMES*^*HIGH*^*CD8*^+^ T memory-exhausted cells (Fig. 2e). These alignments were representative of CD8^+^ T cell-states and signaling orientations observed in ICB-responsive tumors^8,31,47^.

MSS CRC, however, had a completely different alignment structure. Long-term immunosurveillance promiscuously aligned with almost all CD8^+^ T cell subpopulations (Fig. 2f). Over-representation of *EOMES*-signaling, which was indeed associated with the long-term immunosurveillance module here, can potentially limit stem-like phenotype and promote dysregulated exhaustion in CD8^+^ T cells^58,59^. Indeed, we failed to see any clear stem-like module, but we did observe fragmented IR-coding genes’ distributions in MSS CRC *IFNG*^+^CD8^+^ T cells. The inflamed memory-like and tolerogenic c-Maf signaling co-aligned with *EOMES*^*MED*^*TOX*^*MED*^*CD8*^+^ T memory-exhausted and *TOX*^*HIGH*^*CD8*^+^ T memory-exhausted cells (Fig. 2f). This indicated that CD8^+^ T memory-exhausted cells in MSS CRC might have a more mixed inflammatory/tolerogenic orientation than MSI CRC. In addition, all the signaling modules in MSS CRC aligned with a specific *IL17A*^HIGH^ MAIT subset, suggesting their dominant activity in MSS CRC. In conclusion, CD8^+^ T cell signaling hubs in MSI CRC were reminiscent of an ICB-responsive tumor^8,31,47^ whereas those in MSS CRC were largely skewed towards either *EOMES*-dominant signaling or *IL17A*^HIGH^ MAITs, together with mixed inflamed/tolerogenic CD8^+^ T memory-exhausted cells.

### IL17A^HIGH^ MAITs in MSS CRC show TCR reactivity toward microbial riboflavin

MAITs have epitope-specificity restricted to riboflavin intermediates derived from microbes like bacteria, mycobacteria and/or yeasts^60^. MAITs with riboflavin intermediates specific TCR signaling secrete cytokines like IL17 (non-exclusively accompanied by TNF)^60^. Frequently, this is accompanied by wound healing or tissue repair markers like *FURIN*, *HMGB1/2*. In contrast, MAITs activated in a non-TCR specific fashion, favorably secrete IFN-γ and cytolytic factors (*PRF1*, *GZMB*, *FASLG*)^60^. Only in MSS CRC, *IL17A*^+^ MAITs were enriched with the above markers of riboflavin intermediate-specific TCR signaling and tissue repair without general inflammation markers (*TGFB1*, *NFKB1*) (Fig. 2g). Contrastingly, MSI CRC-associated MAITs only showed features of non-TCR specific activation, accompanied by general inflammation markers (Fig. 2g).

Above results, although interesting, originated from transcriptomic signaling and were not confirmatory for the real TCR-specificities. To address this, we used scTCR-seq profiles of these MAITs to directly quantify the TCR-specificity toward riboflavin intermediates. Herein, we accessed published, functionally validated, MAIT-associated TCR α/β-chain sequences specific for riboflavin intermediates^61^ that were then aligned with scTCR sequences of MAITs derived from MSI (Supplementary Fig. S4a, c) or MSS (Supplementary Fig. S4b, d) CRC patients. As hypothesized, the *IL17A*^*HIGH*^ MAITs from MSS CRC patients had the highest alignment scores for riboflavin intermediate-specific TCR sequences (Fig. 2h), but this was not the case for *IFNG*^*HIGH*^ MAITs in MSI CRC (Fig. 2h). MSS CRC clearly showed preference for *IL17A*^HIGH^ MAITs specific for microbe-derived riboflavin intermediates.

### MSI and MSS CRC *IFNG*^+^CD8^+^ T cells show contrasting TCR vs bystander activation

MSS CRC CD8^+^ T cells showed *EOMES*-dominant signaling with mixed inflamed/tolerogenic phenotype which could be indicative of either bystander-activation or TCR-activation^62–64^. To objectively delineate which activation type was applicable to MSI and MSS CRC, we utilized the previously published genetic signatures for TCR-activation and bystander-activation in T cells^65^ (Supplementary Table S3). We visualized the Spearman’s correlation density distributions of *IFNG*^+^CD8^+^ T cells with bystander-activation signature on the *x* axis and TCR-activation signature on the *y* axis (Fig. 2i). Then, we used the normal-adjacent colon tissue-associated *IFNG*^+^CD8^+^ T cells to create a spatial reference point for the population’s distribution center (marked as ‘x’) as well as a 4-quadrant system for differential signature orientation (Fig. 2i). This allowed us to investigate the non-tumoral vs tumoral orientation of the bystander and TCR-activation signatures. Relative to the reference point, MSI CRC *IFNG*^+^CD8^+^ T cells split into two distinct subpopulations: TCR-activation^LOW^bystander-activation^LOW^ and TCR-activation^HIGH^bystander-activation^LOW^ (Fig. 2i). Whereas in case of MSS CRC, *IFNG*^+^CD8^+^ T cells split into three mixed subpopulations: TCR-activation^LOW^bystander-activation^LOW^, TCR-activation^HIGH^bystander-activation^LOW^ and TCR-activation^LOW^bystander-activation^HIGH^ (Fig. 2i). However, the TCR-activation^HIGH^bystander-activation^LOW^ subpopulation was higher in MSI CRC, while the TCR-activation^LOW^bystander-activation^HIGH^ subpopulation was higher in MSS CRC (Fig. 2i).

To validate this, we pursued gene-set enrichment analysis (GSEA) on REACTOME pathways of the TCR-activation^HIGH^ and/or bystander-activation^HIGH^ subpopulations of *IFNG*^+^CD8^+^ T cells. In line with above observations, MSI CRC *IFNG*^+^CD8^+^ T cells enriched for pathways related to TCR activation coupled with IFN-γ/PD-1 signaling, TCR signaling supportive activation-cascade, memory/progenitor-like metabolic profile (*OXPHOS*/TCA cycle)^66^, and immuno-regulatory signaling (Supplementary Fig. S4e). Conversely, MSS CRC *IFNG*^+^CD8^+^ T cells enriched for a huge diversity of pathways including mixed inflammatory (multi-faceted *NFkB* signaling, *IL1* signaling)^67^ or tolerogenic (*TGFβ* signaling)^68^ phenotypes along with TCR signaling, immuno-regulation, type I/II IFN-signaling, PD-1-signaling, proliferative metabolism (glycolysis)^66^ and cellular stress (Supplementary Fig. S4f). Two bystander-like signaling pathways were also observed, i.e., antiviral responses and mycobacterium infection (Supplementary Fig. S4d)^34,64,69^. Thus, MSI CRC *IFNG*^+^CD8^+^ T cells showed higher TCR-activation while MSS CRC *IFNG*^+^CD8^+^ T cells showed heterogeneous inflammatory and tolerogenic characteristics along with higher bystander-like activation.

### TCR repertoire of *IFNG*^+^CD8^+^ T cells in MSS CRC shows low diversity and viral specificity

To ‘functionally’ validate the above transcriptomic observations on the level of TCR repertoire we used a customized Scirpy computational workflow^70^ to perform repertoire mapping as well as antigen-specificity predictions on scTCR-seq profiles of CRC CD8^+^ T cells. We defined TCR clonotypes based on shared *CDR3* sequences, to delineate clusters of T cells with identical epitope or antigen-specificities. These analyses identified a considerable diversity of TCR clonotype clusters in both MSI (Supplementary Fig. S5a) and MSS (Supplementary Fig. S5b) CRC. We used these TCR sequences to query The Immune Epitope Database (IEDB) and annotated them for their predicted epitope specificities using pairwise sequence alignments of *CDR3* amino acid sequences. IEDB hosts experimentally validated data on pairs of human TCR sequences and their known antigen or epitope specificities^71^. These efforts culminated into a categorization of predicted antigen-specificities into broad ‘TCR families’ based on existing IEDB annotations^71^ or annotations from biomedical literature^72^. Such families included viral antigens, shared-cancer antigens, self-antigens, and bacterial antigens (a full list of predicted epitope identities were shown in Supplementary Table S5). Moreover, there were several tumoral TCRs that could not be mapped to any epitope in IEDB, but they showed tumor specificity on account of extremely low overlaps with normal adjacent tissue associated TCRs (Supplementary Fig. S5c). Hence, we annotated these as ‘tumor exclusive TCRs’.

**Fig. 3:**
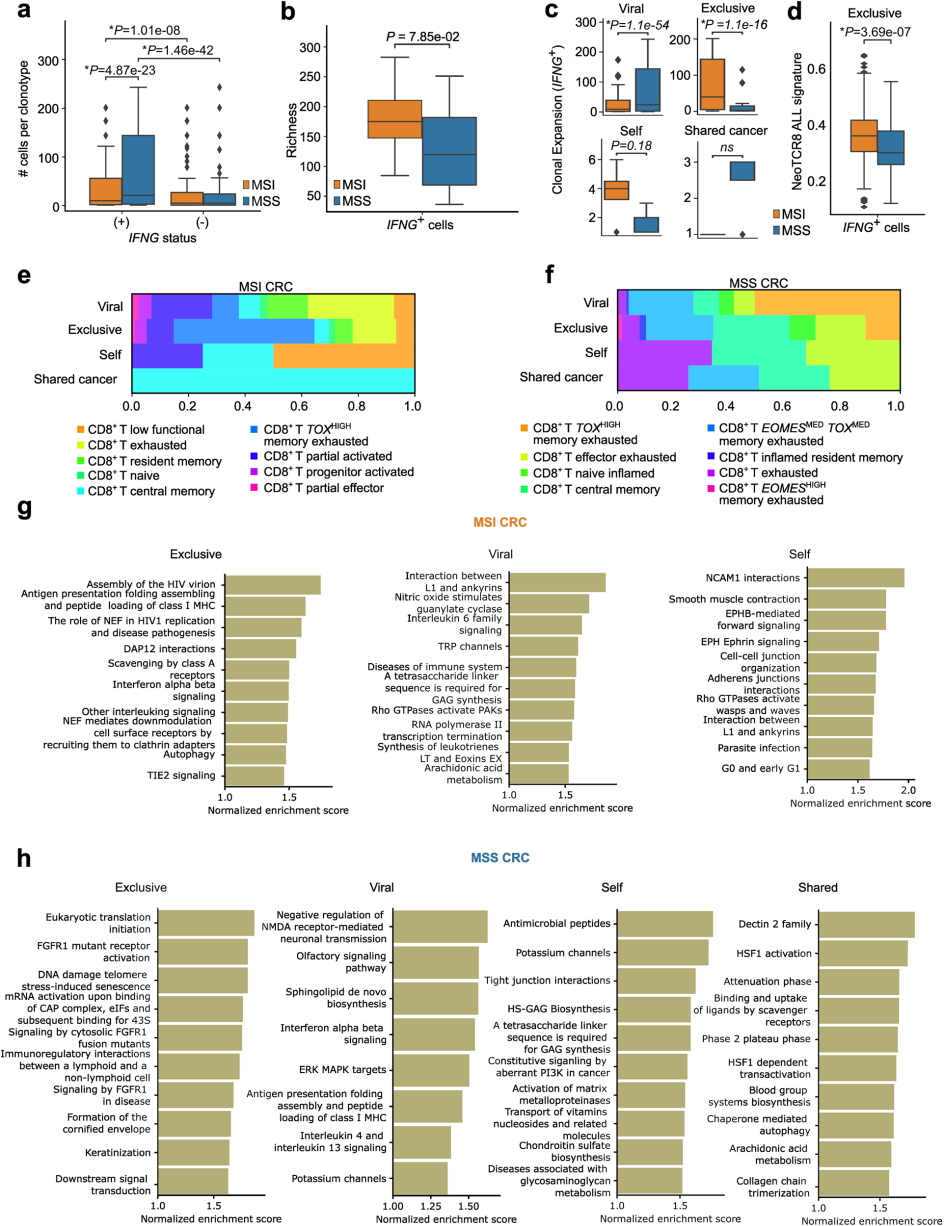
Single-cell TCR sequencing analysis. **a** Boxplots indicating clonotype expansion of the 1753 clonotypes in 7842 CD8^+^ T cells MSI (IFNG^+^: 2338 CD8^+^ T cells, IFNG^–^: 2171 CD8^+^ T cells) and MSS (IFNG^+^: 1611 CD8^+^ T cells, IFNG^–^: 1722 CD8^+^ T cells) across IFNG positivity status. Statistical testing computed using independent *t*-test, *P* value threshold < 0.05). **b** Clonal richness (Chao1 richness estimate) (*y* axis) of the *IFNG*^+^CD8^+^ T cells in these MSI and MSS samples (statistical testing computed using independent *t*-test, *P* value threshold < 0.05). **c** Clonal expansion of *IFNG*^+^CD8^+^ T cells (*y* axis) grouped by predicted antigen epitope groups for viral (MSI: 1865, MSS: 1474), exclusive (MSI: 468, MSS: 130), self (MSI: 4, MSS: 3), and shared cancer antigens (MSI: 1, MSS: 4) (statistical significance calculated using two-sample independent *t*-test, *P* value threshold < 0.05). **d** Mean of binarized expression of NeoTCR8 signature (Supplementary Table S3) genes (*y* axis) for the 3949 *IFNG*^+^CD8^+^ T cells for clonotypes annotated as exclusive based on predicted antigen epitope (statistical significance calculated using independent *t*-test, *P* value threshold < 0.05). **e**, **f** Proportional counts of single cell cluster annotations (see color legend) for *IFNG*^+^CD8^+^ T cells (*x* axis), aggregated by their respective predicted antigen epitope group (*y* axis) in MSI (**e**) and MSS (**f**) CRC samples. **g**, **h** Bar plots visualizing the top pathway terms found to be enriched using GSEA using the Reactome database for each predicted antigen epitope group in MSI (**g**) and MSS (**h**). Terms are sorted based on descending normalized enrichment score.

*IFNG*^+^CD8^+^ T cells accounted for almost all the TCR clonotype expansion (defined as the number of CD8^+^ T cells per TCR clonotype) in MSI/MSS CRC, rather than *IFNG*^−^CD8^+^ T cells (Fig. 3a), thereby supporting our *IFNG*^+^CD8^+^ T cell-centric approach. MSS CRC *IFNG*^+^CD8^+^ T cells showed higher clonotype expansion than MSI CRC *IFNG*^+^CD8^+^ T cells (Fig. 3a). However, TCR polyclonality or diversity (using Chao1 richness estimator for TCR Richness)^73,74^ was relatively higher in MSI CRC *IFNG*^+^CD8^+^ T cells, albeit non-significant (Fig. 3b). High TCR expansion despite low TCR diversity in MSS CRC *IFNG*^+^CD8^+^ T cells could be indicative of low tumoral antigenic diversity^75^.

To better establish this, we categorized TCR clonotype expanders based on TCR families of predicted antigen-specificities. The majority of clonotype expanders were composed of TCRs predicted to recognize viral antigens^76,77^ (Fig. 3c) followed by exclusive TCRs (Fig. 3c). This was in line with previous analyses where most human tumor-associated TCRs had viral-specificities^76^. Remarkably, almost all the TCR clonotype expanders amongst MSS CRC *IFNG*^+^CD8^+^ T cells showed specificity to viral antigens (Fig. 3c). In addition, TCR specificity to another type of bystander antigens such as bacterial antigens (of mycobacterial-origin; Supplementary Table S5), was also predicted exclusively in MSS CRC (Supplementary Fig. S5d), albeit for few clones of *IFNG*^−^CD8^+^ T cell-state only. A significant amount of TCR clonotype expanders in MSI CRC *IFNG*^+^CD8^+^ T cells possessed exclusive TCRs (Fig. 3c). However, due to the extremely sparse number of CD8^+^ T cells annotated for shared or self-antigens, strong conclusions cannot be drawn about their physiological roles.

Considering the high neoantigen load in MSI CRC^3^, we wondered if these T cells could possess neoantigen-reactivity. Hence, we analyzed a previously published neoantigen-reactive TCR-activation signature for CD8^+^ T cells (NeoTCR8)^78^. Amongst CD8^+^ T cells with exclusive TCRs, MSI CRC *IFNG*^+^CD8^+^ T cells showed a higher NeoTCR8-signature, significantly more than their MSS CRC counterparts (Fig. 3d). Finally, few *IFNG*^+^CD8^+^ T cells were predicted to possess shared-cancer antigen (Fig. 3c) or self-antigen specific (Fig. 3c) TCRs, showing a higher expansion in MSS CRC or MSI CRC respectively, albeit non-significant. Thus, MSS CRC CD8^+^ T cells showed low TCR diversity and largely viral TCRs, whereas MSI CRC CD8^+^ T cells showed high TCR diversity and superior enrichment of exclusive TCRs with putative neoantigen-reactive genetic footprint.

### MSI CRC CD8^+^ T cells with exclusive TCRs show immunogenic features and *TOX*^HIGH^ memory-exhaustion, which contrasts with MSS CRC

Next, we aligned the TCR antigen/epitope-specificity predictions with subpopulation phenotypes in *IFNG*^+^CD8^+^ T cells. In MSI CRC, the exclusive TCRs were dominantly expressed by *TOX*^HIGH^CD8^+^ T memory-exhausted cells (Fig. 3e). MSI CRC CD8^+^ T cells with predicted viral TCR were a mixture of several subpopulations (Fig. 3e). Finally, TCRs predicted to bind self-antigens or shared-cancer antigens were dominant in CD8^+^ T low-functional or CD8^+^ T central-memory cells, respectively (Fig. 3e), although this may be inconclusive due to the sparse number of cells. In MSS CRC, the exclusive TCRs as well as TCRs predicted to bind self-antigens or shared cancer antigens were linked to a mixture of CD8^+^ T subpopulations (Fig. 3f), albeit with extremely sparse numbers. However, the TCRs predicted to bind viral antigens in MSS CRC were primarily present in *TOX*^HIGH^CD8^+^ T memory-exhausted cells (Fig. 3f). Thus, depending on MSI or MSS status, *TOX*^HIGH^CD8^+^ T memory-exhausted cells showed different TCR-specificities such as exclusive TCRs in MSI vs viral TCRs in MSS.

To understand if the exclusive TCRs associated with tumor-reactive exhaustion markers, we analyzed the population sizes of all cells with expression of known IR-coding genes. IR-coding genes were prioritized because low expression of at least one IR, CD39 (coded by *ENTPD1*) in CRC CD8^+^ T cells defined a lack of tumor-relevant chronic antigen stimulation and bystander-like phenotype^77^. *IFNG*^+^CD8^+^ T cells with exclusive TCRs had larger populations of cells with expression of almost all IR-coding genes in MSI, rather than MSS, CRC (Supplementary Fig. S5e). MSS CRC *IFNG*^+^CD8^+^ T cells with exclusive TCRs had an extremely low number of cells positive for *ENTPD1* as well as a very small number of both *PDCD1*^+^*HAVCR2* ‘pre-exhausted’ and *PDCD1*^+^*HAVCR2*^+^ ‘exhausted’ profiles, compared to MSI CRC (Supplementary Fig. S5e). This implied that exclusive TCRs in MSS CRC did not create a typical chronic exhaustion profile. There were no notable differences in IR-coding gene-positive populations between MSI and MSS CRC for *IFNG*^+^CD8^+^ T cells with TCRs predicted to bind viral antigens (Supplementary Fig. S5e).

Finally, we did an *IFNG*^*+*^ vs *IFNG*^–^CD8^+^ T cells’ GSEA using REACTOME pathway terms per TCR antigen-specificity family. MSI CRC *IFNG*^+^CD8^+^ T cells with exclusive TCRs enriched for various immunogenic pathways related to IFN signaling, antigen presentation, and antiviral immune response (Fig. 3g). Contrastingly, in MSS CRC, *IFNG*^+^CD8^+^ T cells with exclusive TCRs enriched for pathways related to either immunoregulation, wound healing (i.e., FGFR1 signaling^79^) or cellular stress/senescence (Fig. 3h). *IFNG*^+^CD8^+^ T cells possessing viral TCRs also had relatively distinct features (Fig. 3g, h), i.e., while MSI CRC cells with viral TCRs enriched for largely metabolic pathways yet MSS CRC cells with viral TCRs enriched for either immunogenic pathways like IFN signaling, antigen presentation or IL4/IL13 immunosuppression. MSS or MSI *IFNG*^+^CD8^+^ T cells with self-antigen or shared cancer-antigen oriented TCRs did not show any dominant immunological pathways although self-antigen oriented TCRs showed some tendency to enrich anti-microbial/anti-parasitic pathways (Fig. 3g, h). In conclusion, MSI CRC CD8^+^ T cells with exclusive TCRs showed chronic exhaustion and pro-immunogenic features and connected to *TOX*^*HIGH*^CD8^+^ T memory-exhausted cells. However, MSS CRC CD8^+^ T cells showed less tumor-reactive exhaustion and more immunoregulatory/pro-stress responses in exclusive TCR context.

### CD8^+^ T cell features exhibit contrasting multi-layered clustering in MSI vs MSS CRC

**Fig. 4:**
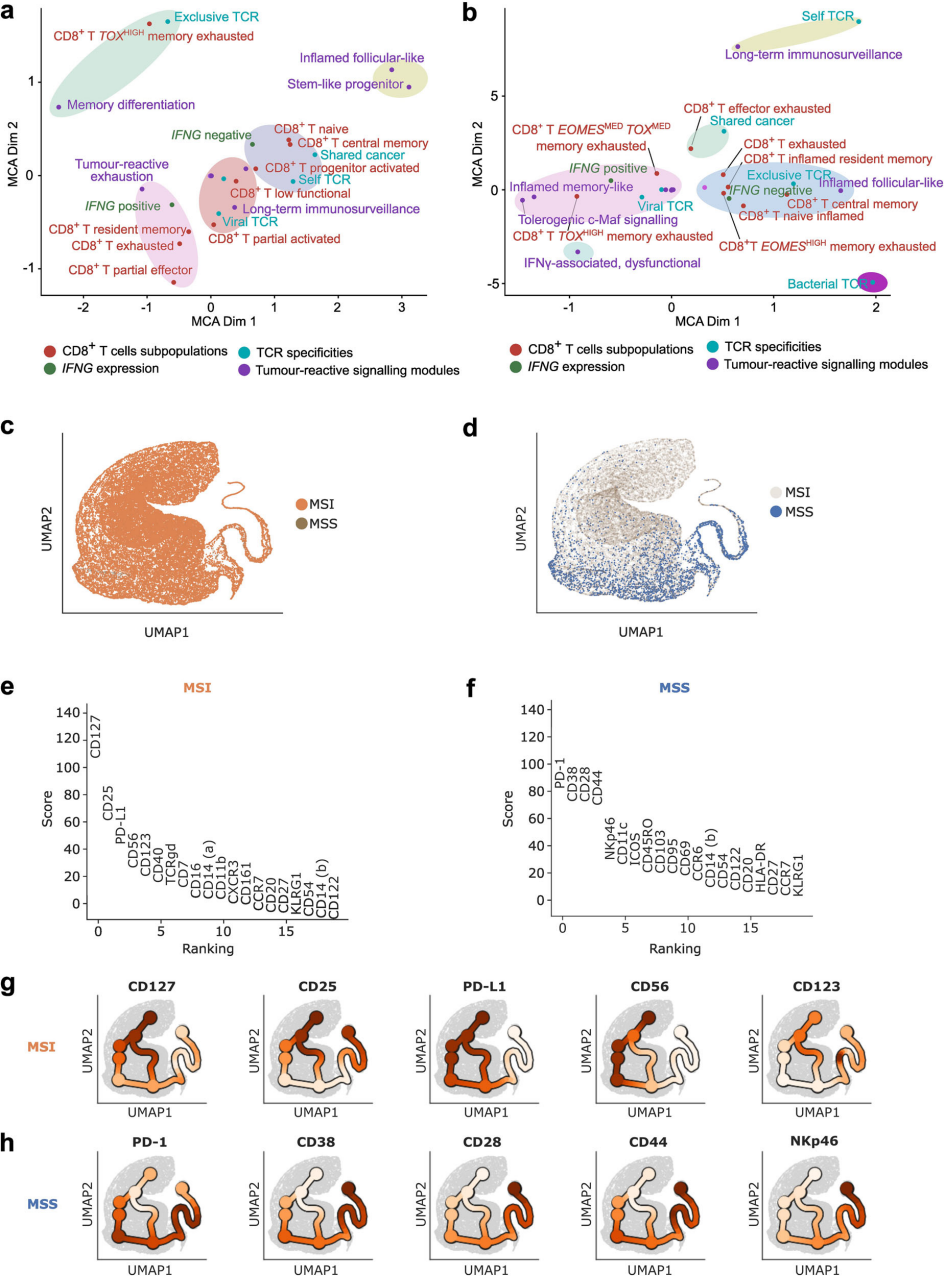
Multiple correspondence analysis and CyTOF validation. **a**, **b** Multiple Correspondence Analysis (MCA) on the various annotations for CD8^+^ T cells produced in this study for MSI (**a**) and MSS (**b**). K-Means graphical distance clustering was used (circles). Unlabelled data points show the low signal categories of binarized factors (color) (see Supplementary Material & Methods). **c**, **d** UMAP representation of 39,457 CD8^+^ T cells (1,547,841 cells total) in a publicly available CyTOF dataset (FR-FCM-Z24H) spanning 30 CRC patients post quality control (31 prior to filtering). Results for the 8 MSI (**c**) and 22 MSS (**d**) patients are shown, covering 35,354 and 3410 CD8^+^ T cells respectively. **e**, **f** Differential gene expression analysis using Scanpy’s ‘rank_genes_groups’, indicating genes upregulated in MSI vs MSS (**e**) or MSS vs MSI (**f**), using two-sided *t*-tests with Benjamini-Hochberg correction for multiple testing (significance threshold *P* < 0.05). **g**, **h** Trajectory inference using scFates on the CyTOF object, post loading and pre-processing with Pytometry (https://github.com/buettnerlab/pytometry) and Scanpy. Diffusion maps were generated using Palantir. Expression of the top 5 most discriminative genes in MSI vs MSS (**g**) and MSS vs MSI (**h**) along the trajectory are shown. The UMAP space is identical to panels (**c**) and (**d**) in this figure.

The above analyses emphasized the importance of at least four transcriptomic features across CRC CD8^+^ T cells, i.e., CD8^+^ T cell sub-populations, *IFNG* expression, predicted TCR antigen-specificities and tumor-reactive signaling modules. However, the above analyses were supervised as reductionist approaches. Hence, we wondered if (and how) these transcriptomic features cross-arranged as de novo clusters in an unsupervised space. To address this, we performed a de novo unsupervised clustering analysis between the above four features with a multiple correspondence analysis (MCA) using categorical values from CD8^+^ T cell sub-populations, *IFNG* expression, predicted TCR antigen-groups, and tumor-reactive signaling modules. This projection arranged CD8^+^ T cells from MSI CRC and MSS CRC into several distinct groups that were covered by five clusters (Fig. 4a) and six clusters, respectively using K Means (Fig. 4b). Intriguingly, the composition of these clusters conformed to the above observations. For instance, in MSI CRC the exclusive TCRs co-clustered with memory differentiation module and *TOX*^HIGH^CD8^+^ T memory-exhausted cells (Fig. 4a), while viral TCRs were aligned with more long-term immunosurveillance and low functional or partially activated CD8^+^ T cells. *IFNG*^+^ status was associated with exhaustion or resident memory-characteristics whereas *IFNG*^–^ status associated with self/shared-cancer TCRs or naive/un-activated status of CD8^+^ T cells. Stem-like progenitor and inflamed follicular-like modules showed distinct co-clustering.

Contrastingly, in MSS CRC, the exclusive TCRs simultaneously co-clustered with *IFNG*^–^ status and naive, inflamed, or exhausted CD8^+^ T cell-features as well as *EOMES*^HIGH^CD8^+^ T memory-exhausted cells (Fig. 4b). The functionally crucial *IFNG*^+^ status preferably co-clustered with viral TCRs, *TOX*^HIGH^ or *EOMES*^*MED*^*TOX*^*MED*^CD8^+^ T memory-exhausted cells, and inflamed memory-like or tolerogenic c-Maf signaling. Interestingly, the long-term immunosurveillance module that dominated the MSS CRC CD8^+^ T cells, preferably co-clustered with self TCRs. In conclusion, CD8^+^ T cells in MSI CRC and MSS CRC show highly distinct ‘multi-layered’ clustering of their functionally crucial transcriptomic features.

### MSI CRC CD8^+^ T cells show distinct proteomic effector memory features

Since our study largely hinged upon single cell transcriptomic data, we felt it was essential to validate our observations on proteomic level. To this end, we accessed a publicly available CyTOF dataset of CD8^+^ T cells (1,547,841 cells total, with 39,457 CD8^+^ T cells remaining after quality control) derived from 8 MSI and 22 MSS patients (some patients were excluded due to quality control)^80^. On this dataset, we performed a trajectory analysis which exposed either a largely exclusive trajectory for MSI CRC CD8^+^ T cells or an overlapping trajectory between MSI and MSS CRC CD8^+^ T cells (Fig. 4c, d). Herein, an MSI vs MSS CRC analyses for differential surface marker enrichment (Fig. 4e, f) showed that MSI CRC CD8^+^ T cells pre-dominantly exposed markers of memory phenotype (CD127^81^), effector memory or polyclonal TCR phenotype (CD25^82^), immunoregulation (PD-L1^83^), T cell activation (CD56^84^), and cytokine signaling (CD123^85^) (Fig. 4e, g). In contrast, MSS CRC CD8^+^ T cells exposed a lot more contradictory inflammatory markers, i.e., immuno-regulation (PD-1^31^), suppressor of T cell activity (CD38^86^), T cell activation (CD28, CD44^87,88^) and unconventional T cells (NKp46^89^) (Fig. 4f, h). Altogether, we observed that MSI CRC CD8^+^ T cells expose more effector memory-like surface markers, while MSS CRC CD8^+^ T cells show heterogeneous or contradictory surface markers of inflammation.

### CD8^+^ T cell dynamics operate as patient-dependent strata within MSI and MSS CRC

The tumor-reactive signaling modules were particularly proficient at capturing the contrasting CD8^+^ T cell-dynamics in MSI vs MSS CRC. However, considering how immunotherapy responses operate^90^, these dynamics were most likely not limited to MSI or MSS CRC contexts, but extendable to patient-dependent variations (Supplementary Table S1). Hence, we investigated if the 22 MSI CRC and 38 MSS CRC patients (due to thresholding) can be broadly stratified on the tumor-reactive signaling modules (using hierarchical clustering of per patient means of the tumor-reactive modules), and how such stratification aligns with other annotations like CD8^+^ T cell subpopulations, TCR-to-bystander activation status, and IRs-coding genes’ expression.

**Fig. 5:**
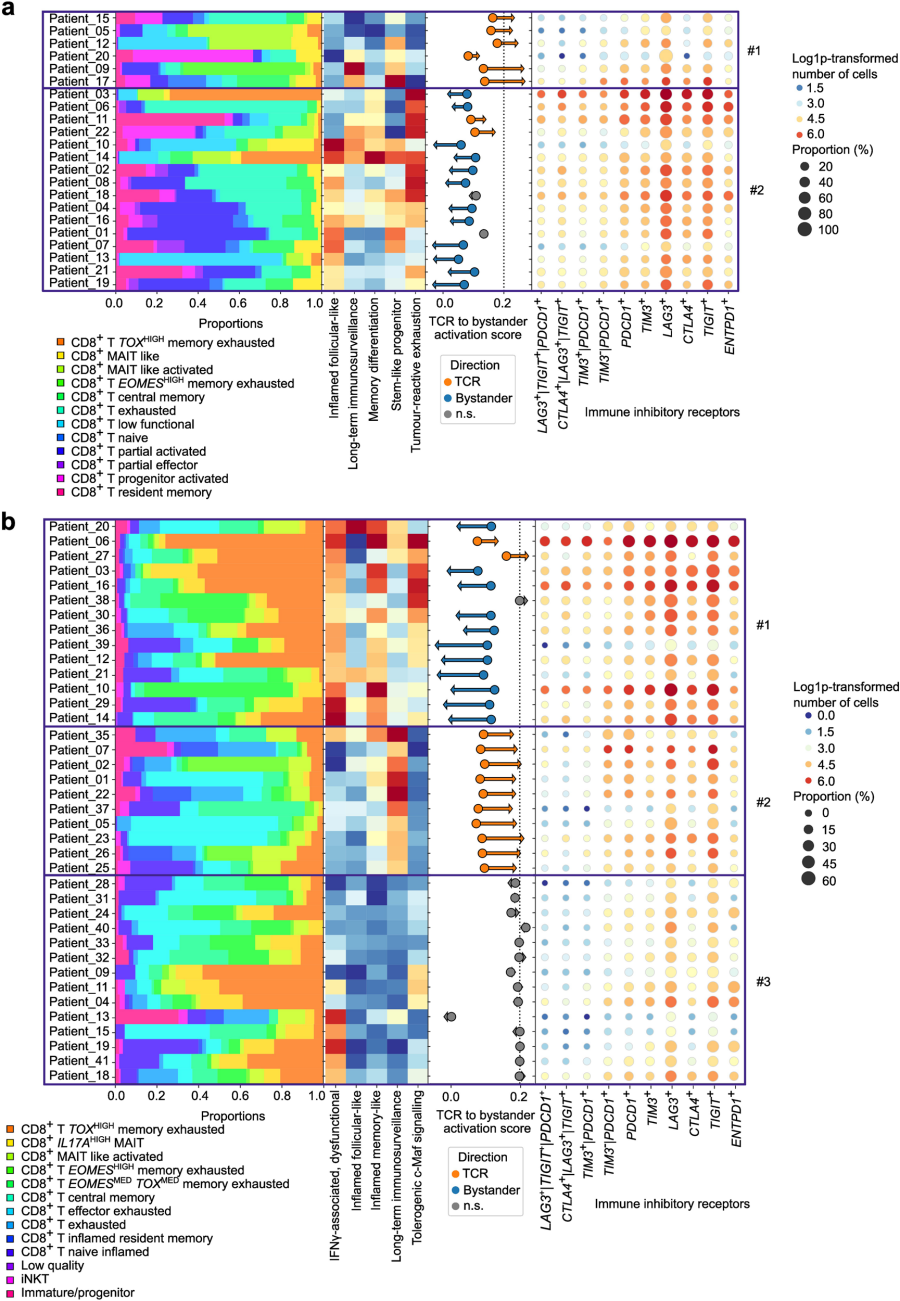
Stratification of MSI and MSS CRC patients based on scRNA-seq signaling modules. **a**, **b** Visual representation of single cell patient subgroup detection. CD8^+^ T cell population counts per patient are shown as relative proportions (stacked bar plots). For each patient, the ratio of bystander activation signature expression to TCR activation expression is represented as a dot at the basis of an arrow (Supplementary Table S3). Arrows indicated the difference in median correlation between TCR activation and bystander activation when compared to the corresponding resting state. Dot plots on the right-hand side indicated the number of cells positive for expression of important immune-inhibitory receptor (IR) genes when compared to a threshold value. Vertical lines “|” in gene names indicate double positive cells for the genes indicated. The size of the dots represents the proportion of this population compared to the total CD8^+^ T cell pool. Heatmaps represent the expression of the tumor-reactive signaling modules. **a** MSI CRC patients were stratified into roughly two tumor-associated clusters (indicated on the right-hand side with a number, containing six patients for group 1 and 16 for group 2) using hierarchical clustering on the tumor-reactive signaling module scores (central heatmap). **b** MSS CRC patients were roughly stratified into three tumor-associated clusters using hierarchical clustering on the expression of the tumor-reactive signaling modules (heatmap), as indicated on the right-hand side with a number; 1: 14 patients, 2: 10 patients, 3: 14 patients. Data for three patients was not available due to thresholding.

In MSI CRC, the tumor-reactive signaling modules stratified the patients into two clusters (Fig. 5a). The cluster #1 preferentially showed stem-like progenitor module, alone or together with long-term immunosurveillance (Fig. 5a). Cluster #2 captured patients with mixed inflammatory status, led by either tumor-reactive exhaustion and/or inflamed follicular-like modules (Fig. 5a). To get an idea of how TCR activation and bystander activation trended, we used 3 signatures available from MSIGDB (GSE13738): TCR_VS_BYSTANDER_ACTIVATED_CD4_TCELL_DN (signature #1), RESTING_VS_BYSTANDER_ ACTIVATED_CD4_TCELL_DN (signature #2) and RESTING_VS_TCR_ACTIVATED_CD4_TCELL _DN (signature #3). We then calculated the Spearman correlations between signature #1 and #2 (R_SPB_, marking bystander activation), as well as between signature #1 and signature #3 (R_SPA_, marking TCR activation). The difference of the median values of R_SPA_ and R_SPB_ was the measure used as representative of directionality between TCR activation vs bystander activation. We found that this directionality metric was increased in cluster #1 when compared to cluster #2 (Fig. 5a).

For MSS CRC, the situation was more complex. The tumor-reactive signaling modules achieved an heterogenous division of the patients into three clusters (Fig. 5b). Cluster #1 showed a mixed inflammatory status, led by largely overlapping co-enrichments of IFN-γ-associated dysfunctional, inflamed memory-like and tolerogenic c-Maf signaling modules (Fig. 5b). Cluster #2 demonstrated long-term immunosurveillance, while cluster #3 showed signs of ‘immune silence’, as it showed low signal in most of the modules (Fig. 5b). The directionality score between TCR activation and bystander activation per patient was consistently higher in cluster #2 rather than cluster #1 while it was non-directional in cluster #3 (difference < 0.2) (Fig. 5b). The pattern for the ratio of TCR-to-bystander activation signature did not align with IR-coding gene’s e.g., cluster #1 which showed high TCR-to-bystander activation signature ratio but a lower number of cells positive for IR-coding gene expression when compared to clusters #2 or #3 (Fig. 5b). Various patients in cluster #1 showed strong signal for *TOX*^HIGH^CD8^+^ T memory-exhausted cells, a trait that they share with cluster #3 (Fig. 5b). Overall, there was a sizable patient-to-patient variation in MSI or MSS CRC for CD8^+^ T cell features. MSI CRC patients bifurcated into more homogeneous clusters with relatively consistent CD8^+^ T cell dynamics, whereas MSS CRC patients exhibited heterogeneous clusters with contradictory CD8^+^ T cell dynamics.

### CD8^+^ T single cell-derived tumor-reactive signaling modules can stratify MSI and MSS CRC patients’ bulk tumor transcriptomes

We wondered if the tumor-reactive signaling modules could also stratify CRC patients into a prognostic or predictive immune subtype classification (ISC) system. Such a prognostic or predictive biomarker framework on the single cell level is currently almost irrelevant, as datasets integrating clinical patient survival or ICB-responses are still mainly comprised of bulk tumor transcriptomes. Interestingly, a network topology-based analysis for network expansion (WEB-based Gene SeT AnaLysis Toolkit)^41^ showed that the genes constituting the tumor-reactive signaling modules on single cell CD8^+^ T cell level, also pre-dominantly connected to lymphocytes or T cell-relevant pathways in TCGA CRC dataset’s bulk tumor transcriptomes (Supplementary Fig. S6a).

**Fig. 6:**
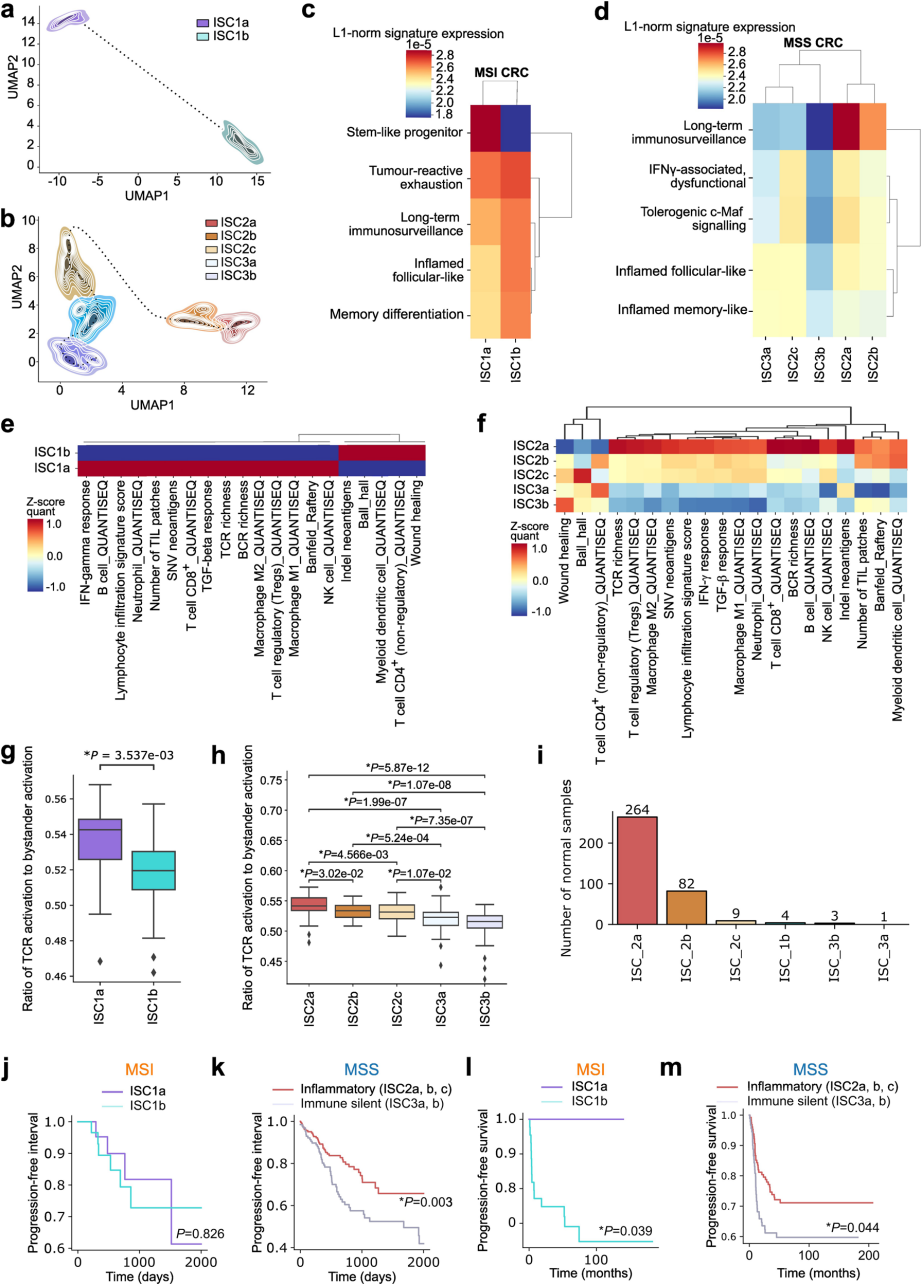
Stratification of MSI and MSS CRC patients’ bulk tumor transcriptomes. **a**, **b** Density maps for 57 MSI (**a**) and 317 MSS (**b**) primary colorectal tumor samples in TCGA. Patients were aggregated in clusters using agglomerative clustering on patients in the manifold space into two and three groups for MSI and MSS, respectively. The manifold space was defined by patient scores across each of the individual single cell-derived tumor-reactive signaling modules. The resulting two MSI and MSS bulk RNA-seq clusters were then referred to as ISC (immune subtype classification) clusters. **c**, **d** L1-normalised expression per patient of metagenes representing the tumor-reactive signaling modules (*y* axis) for each ISC cluster (*x* axis) in the TCGA MSI (**c**) and MSS (**d**) patient cohorts. **e**, **f** Z-score normalized scores of immune parameters or gene signature expression across the ISC clusters (*x* axis) in TCGA. Cell populations were inferred using the quanTIseq deconvolution. Other antigenicity parameters (Indel/SNV neoantigens, TCR/BCR Richness), immune-landscape signatures and H&E tumor image-based deep learning parameters for tumor-infiltrating lymphocytes (TILs) (*y* axis) are also represented. **g**, **h** Ratio of TCR activation to bystander activation signature expression in the 374 primary CRC samples in TCGA across ISC clusters for MSI (**g**) and MSS (**h**). **i** Barplots indicating number of normal samples per ISC category (363 normal samples in total) as called by the ISC classifier (exact numbers listed above bar). ISC1a was not called as the most likely label in any of the normal samples. **j**, **k** Kaplan–Meier curves indicating progression-free interval in TCGA for MSI (ISC1a: 24 patients, ISC1b: 33 patients) (**j**) and MSS (Inflammatory: 187 patients, Immune silent: 130 patients) (**k**) respectively. **l**, **m** Kaplan–Meier curves illustrating progression-free survival in the independent SIDRA-LUMC cohort for MSI (ISC1a: 11 patients, ISC1b: 46 patients) (**l**) and MSS (Inflammatory: 131, Immune silent: 93) (**m**).

Hence, we investigated if the ten tumor-reactive signaling modules can stratify 374 TCGA MSI^HIGH^ or MSS (MSI-L and MSS) CRC patients, using agglomerative clustering in the MSI or MSS manifold space defined by these modules (Fig. 6a, b). MSI^HIGH^ CRC patients (ISC1) bifurcated into two clusters (ISC1a and ISC1b) (Fig. 6a); wherein ISC1a highlighted specific enrichment of the stem-like progenitor module while ISC1b showed a mixed inflammatory status led by tumor-reactive exhaustion (Fig. 6c). Contrastingly, the MSS CRC patients were distributed over five clusters (ISC2a/2b/2c or ISC3a/3b) (Fig. 6b). Herein, ISC2a, 2b and 2c showed variable enrichment for all modules, thereby signifying a mixed inflammatory status. We therefore labeled ISC2 as mixed inflamed (Fig. 6d). ISC2a/2b were distinguished from ISC2c by the former’s stronger enrichment for the long-lived immunosurveillance module (Fig. 6d). Contrastingly, ISC3a/3b showed a more ‘immune-silent’ phenotype owing to either extremely low (ISC3a) or altogether negligible presence (ISC3b) of all modules (Fig. 6d). Moreover, the ISC framework represented a unique classification of CRC patients, rather than repetition of other existing systems, because the ISC clusters did not consistently align with any of the widely used epithelial-driven CMS subgroups (Supplementary Fig. S6b). Thus, the CD8^+^ T cells’ transcriptome-derived tumor-reactive signaling modules proficiently stratified CRC patients’ bulk tumor transcriptomes within a new ISC framework.

### ISC clusters show distinct tumor immune landscape across MSI vs MSS CRC

To benchmark this classification system on pan-tumor level, we examined the clinico-immunological features of ISC clusters. We performed normalized scoring across TCGA ISC clusters (Supplementary Table S6) for transcriptomic vs pathological image-based immune features: immune cell quantification from immune-deconvolution (quanTIseq)^91^, antigenicity parameters (Indel/SNV neoantigens, TCR/BCR Richness; TCGA data)^73^, and immune-landscape signatures (TCGA data) vs hematoxylin and eosin (H&E) tumor image-derived deep learning metrics describing behavior of tumor-infiltrating lymphocytes (TILs; TCGA data)^92^. In MSI^HIGH^ CRC patients, ISC1a is associated with almost all immunological features, including TCR Richness (Fig. 6e). ISC1b showed an increased multi-factorial wound healing pathway^31,73^ together with mixed myeloid-lymphoid features and InDel neoantigens (Fig. 6e). In MSS CRC patients, the ISC2 showed higher immunological features, whereas ISC3 (similar to ISC1b in MSI^HIGH^ CRC) mainly showed wound healing and some lymphocytic parameters (Fig. 6f). But, ISC2 had high heterogeneity amongst its three clusters such that ISC2a had the highest signal for the above features followed by ISC2b and ISC2c (Fig. 6f). ISC3a and ISC3b were remarkably similar in their immune-silent phenotype (Fig. 6f).

Interestingly, in MSI^HIGH^ CRC, the ratio for TCR-to-bystander activation signature was significantly higher in ISC1a than ISC1b (Fig. 6g); while in MSS CRC, this ratio was highest in ISC2a and from thereon kept decreasing like a gradient until it reached the lowest points in ISC3a/3b (Fig. 6h). On single cell level, we frequently observed quantitative vs qualitative disconnect between CD8^+^ T cell features, especially for TCR-relevant signaling in MSS CRC. Hence, we did a qualitative analysis of TCR diversity (TCR Richness metric) which was correlated to various published immunogenicity-related genetic signatures of neoantigen-reactive CD4^+^ T cells (NeoTCR4) or CD8^+^ T cells (NeoTCR8)^78^, TCR or bystander activation signatures^65^ and a T cell-inflamed gene expression profile signature (GEP)^93^ in bulk TCGA transcriptomes. In MSI^HIGH^ CRC, ISC1a showed more correlation than ISC1b between these signatures and TCR Richness (Supplementary Fig. S6c). However, for MSS CRC, ISC2c showed the highest correlation between the above signatures and TCR Richness (Supplementary Fig. S6c).

Above data exposed the high heterogeneity and qualitative vs quantitative disconnections in TCR-relevant features of MSS CRC. Based on our single cell data, we wondered if this indicated a more bystander or tumor-unspecific origin for ISCs in MSS CRC, i.e., driven by tumoral (gut) microbiome and/or colon tissue-specific ‘self’ features^94^. For tumoral microbiome analyses, we used the viral or bacterial call-outs predicted from non-human reads in TCGA CRC data via KRAKEN^95^. However, these tumoral virome and bacteriome features did not reliably predict the ISCs (Supplementary Fig. S7a, b). This indicated that the ISCs were probably not driven by tumoral microbiome. Instead, we found that these tumor microbiome features strongly discriminated between MSI and MSS CRC (Supplementary Fig. S7c). Within the top ranked features herein, we found several bacterial genera that are suggested to associate with CRC pathogenesis, e.g., *Fusobacterium*^96^, *Paeniclostridium*^97^, *Bartonella*^98^ and viruses such as alpha-papillomavirus^99^ (Supplementary Fig. S7d).

Finally, to investigate the role of colon tissue-specific ‘self’ features, we performed ISC prediction on a joint batch-corrected TCGA-GTEx (Genotype-Tissue Expression) dataset comprising of GTEx-derived normal or non-diseased colon tissue’s transcriptome and TCGA’s CRC tumor transcriptomes for comparison^100^ (Fig. 6i; Supplementary Fig. S7e, f). Remarkably, most of the normal tissue was predicted to be ISC2a or ISC2b, while all other types were more evenly recovered in the tumor tissue (Fig. 6i). Accordingly, the overall classification probability of a normal colon tissue indicated an overwhelming tendency for normal samples to be scored as ISC2a or 2b (Fig. 6i). This suggested that within MSS CRC, the most inflammatory clusters (ISC2a, ISC2b) may not be entirely tumor specific. Thus, our results suggest that ‘self-like’ bystander signals could be highly prevalent in highly inflammatory ISC classes of MSS CRC.

### ISC clusters show prognostic impact in MSI or MSS CRC patients

Next, we pursued prognostic analyses for these ISC clusters in the TCGA CRC cohort. In MSI^HIGH^ CRC, ISC1a showed a non-significant trend toward higher progression-free survival (PFS) than ISC1b (Fig. 6j). In MSS CRC, the ISC2 cluster showed significantly prolonged PFS compared to ISC3 (Fig. 6k). To validate our findings in an independent CRC patient cohort, we tested the prognostic impact of ISC in the recently published SIDRA-LUMC CRC cohort, containing 348 patients (57 MSI and 224 MSS CRC)^101^. Herein, we recovered either better or similar trends for PFS as in TCGA, i.e., ISC1a MSI CRC patients survived significantly better than ISC1b patients (Fig. 6l). Similarly, ISC2 MSS CRC patients also survived significantly longer than ISC3 MSS CRC patients (Fig. 6m).

We further compared the prognostic impact of ISC and CMS systems with a multi-variate Cox Proportional Hazard regression model in both TCGA (Supplementary Fig. S8a) and SIDRA-LUMC (Supplementary Fig. S8b). Here, depending on the availability, we accounted for co-variates like age, gender/sex, and tumor purity. For MSI CRC, ISC1a trended toward positive prognostic impact (primarily in SIDRA-LUMC), however, this was insignificant (*P* = 0.09). For CMS, no clear pattern was visible. In MSS CRC, the differences were noticeably different. ISC2 is associated with significantly better patient prognosis in TCGA (HR = 0.56, *P* = 0.02), with a similar positive trend in SIDRA-LUMC (HR = 0.63, *P* = 0.05). For CMS, CMS2 is associated with significantly better patient prognosis in TCGA (*P* = 0.01), with a similar positive trend in SIDRA-LUMC cohort (*P* = 0.08). However, CMS3 or CMS4 did not significantly predict PFS trends although the latter mostly linked with worse PFS. These trends for prognostic impact of CMS are in-line with published literature^102^ but do not necessarily align with CRC immunogenicity, i.e., while CMS2/4 are supposed to be poorly immunogenic with higher affinity for innate immune responses or general inflammation yet CMS3 is supposed to be highly immunogenic with better adaptive immune responses^102^. Yet, CMS3 does not show clear prognostic impact whereas CMS2/4 show opposite prognostic trends. On the contrary, the ISC2 clearly captures CRC patients with high T cell infiltrates and high inflammation, and these survive better than immune-silent ISC3. Thus, the ISC framework shows promising prognostic value in CRC patients and aligns the CRC T cell infiltration patterns better with prognosis than the CMS framework.

### ISC scoring predicts immunotherapy responses in CRC patients on bulk tumor level

We evaluated if the ISC clusters can show predictive impact for immunotherapy responsiveness. Based on these TCGA ISC subtypes, we built a Random Forest classification model to predict the ISC subtype labels from bulk transcriptome profiles of MSI or MSS CRC patients. We assessed this classification approach in clinical trials, treated with anti-PD-1 and/or anti-CTLA4 ICBs. In the publicly accessible record, there were only 2 clinical trials available with ICB-treatments, tumor transcriptome, and clinical objective response-rates (one each for MSI or MSS CRC)^17,103^. In case of MSS CRC, we pursued a conventional responder (complete response (CR) + partial response (PR)) vs non-responder (stable disease (SD) + progressive disease (PD) together) comparison. In MSI CRC we compared durable responder (CR) vs the rest (PR, SD, PD), because there was a tendency for PR to become PD over time, which was not the case for CR^17^.

**Fig. 7:**
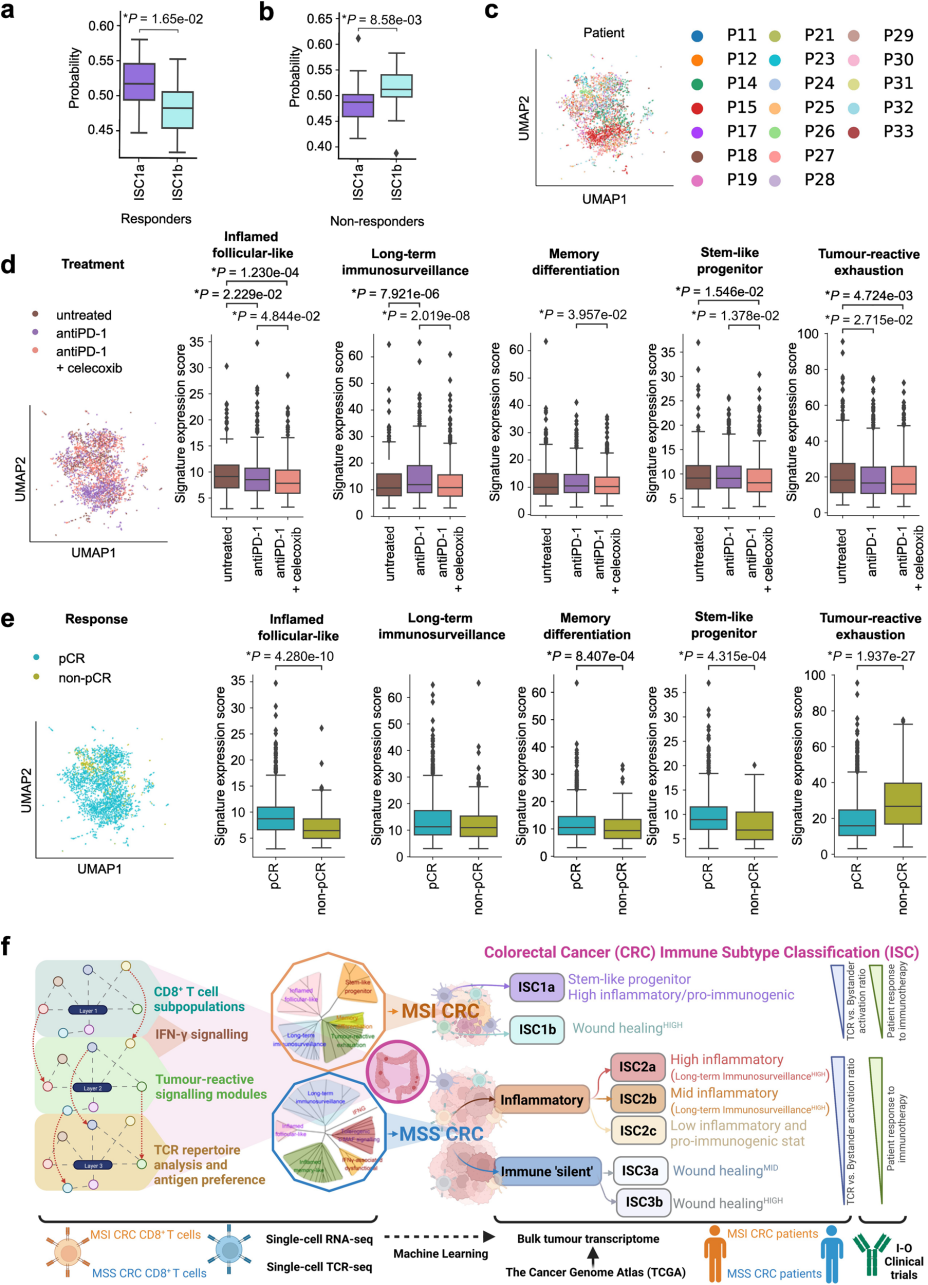
Validation in CRC immuno-oncology clinical trials. **a**, **b** Analysis of the Keynote-177 trial in which MSI CRC patients were treated with anti-PD-1 ICB, using a random forest classifier. Classification probabilities were assigned to the Keynote-177 samples by MSI-specific prediction model that used the TCGA bulk RNA-seq labels and corresponding ISC labels as training input. Results are shown for the 40 samples associated with the five durable responder patients (**a**) and the 33 samples associated with the 11 non-durable responders (**b**). Statistical significance was assessed with Welch’s *t*-test (significance: *P* < 0.05). **c**–**e** Analysis of a longitudinal single cell dataset with 155,397 cells from 19 MSI CRC patients, profiled pre vs post treatment with anti-PD-1 ICB (10 patients) or anti-PD-1+celecoxib (9 patients). Of these 155,397 cells, 3260 could be confidently identified as CD8^+^ T cells, which we subsequently analyzed. **c** UMAP of the individual patients. **d** Analysis of the treatment effects, with UMAP representation and boxplots illustrating metagene expression of the tumor-reactive modules per treatment regimen. **e** Comparison of response categories (pathological complete response or pCR vs non-pCR), with UMAP representation and boxplots illustrating metagene expression of the tumor-reactive modules per patient response. Here, 15 patients showed pCR and 4 patients showed non-pCR. **f** A schematic overview of our study’s approach from single cell data to tumor-reactive signaling modules and how this resulted in the novel Immune Subtype Classification (ISC) system for colorectal cancer (see discussion for details).

In MSI CRC patients treated with anti-PD-1 ICB (KEYNOTE-177 clinical trial), durable responders had significantly higher probability to be called as ISC1a than PR/non-responders (Fig. 7a), whereas for the latter patients the pattern was completely opposite (Fig. 7b). This confirmed that the ISC1a is associated with durable response or CR to anti-PD1 ICB in MSI CRC patients. Of note, in this cohort, multiple regions were transcriptomically profiled per tumor/patient^17^. Although we considered a pooled ISC score per patient, there was some degree of heterogeneity in ISC scores depending on different intra-tumoral regions for the same patient. This was particularly the case for ISC1b (Supplementary Fig. S8c). For samples from MSS CRC patients treated with anti-PD-1 + anti-CTLA4 ICBs together with radiotherapy (NCT03104439)^103^, the only partially responding CRC patient (PR) with tumor transcriptomic data had higher ISC2 score than ISC3, whereas in non-responders (SD + PD) there was no clear trend (Supplementary Fig. S8d). Due to lack of sufficient power, statistical analyses of this MSS CRC cohort were not feasible. This confirmed that our novel CRC ISC framework can be applied as a scoring system to delineate immunotherapy responders, at least in MSI CRC. For MSS CRC, better powered cohorts are urgently needed.

### ISC signaling modules in CD8^+^ T cells predict immunotherapy responses in MSI CRC

The ISCs were driven by CD8^+^ T cell-derived signaling module signatures. To this end, we wondered whether like the ISC scoring, also these signaling modules had the ability to predict immunotherapy responses in CRC patients thereby connecting the ISC concept across both bulk tumor and single cell resolutions. To address this, we accessed single cell profiles of tumoral CD8^+^ T cells from a recently published scRNAseq dataset of 19 MSI CRC patients (GSE205506)^104^ (Fig. 7c), that were either untreated, treated with anti-PD-1 ICB or a combination of anti-PD-1 ICB and celecoxib (NCT03926338) (Fig. 7d). Herein, we compared durable responder (CR) vs the rest (non-CR) (Fig. 7e).

Compared to CD8^+^ T cells from untreated MSI CRC, only the long-term immunosurveillance signaling module showed significant increase in CD8^+^ T cells from anti-PD-1-treated MSI CRC (Fig. 7d). Other modules either remained unchanged or showed mild reductions (Fig. 7d). The most interesting patterns were visible when comparing immunotherapy responder vs non-responder MSI CRC patients. Here, stem-like progenitor and inflamed follicular-like signaling modules were significantly increased in CD8^+^ T cells from immunotherapy responder MSI CRC patients (Fig. 7e). Curiously, increased tumor-reactive exhaustion in CD8^+^ T cells was significantly associated with immunotherapy non-responsive MSI CRC patients, whereas others showed mild differences (Fig. 7e). In conclusion, the stem-like progenitor and inflamed follicular-like signaling modules in CD8^+^ T cells predict durable immunotherapy responses in MSI CRC patients.

## Discussion

Our study shows that CRC CD8^+^ T cells are organized into different layers of subpopulation complexities depending on phenotypic features, IFN-γ signaling, and tumor-reactive signaling modules. The integration of these layers into homogeneous tumor-reactive signaling (in MSI CRC) vs heterogenous inflammatory/tolerogenic signaling (in MSS CRC) is principally driven by TCR diversity and their antigenic/epitope specificities together with complex environmental cues like gut microbiome or colon tissue-specific ‘self-like’ features. Such environmental cues seemed to influence CD8^+^ T cell subpopulation dynamics toward bystander-like activation in low-antigenic milieu like MSS CRC. On the contrary, an antigenic milieu in MSI CRC facilitated dominance of TCR-activation thereby pre-priming the tumor for immunotherapy responsiveness.

In general, while our results did quantitatively align with previously reported differences between MSI and MSS CRC (e.g. quantities of *IFNG*^+^ or *CXC13*^+^CD8^+^ T cells^1^, subtle exhaustion phenotypes^18^), these were too subtle to account for the very big clinico-immunological differences between these subtypes. Instead, we could better highlight these differences on the level of signaling contexts and predicted TCR specificities. For instance, MSI CRC enriched more *IFNG*^+^CD8^+^ T cells than MSS CRC, however, the smaller amounts of *IFNG*^+^CD8^+^ T cells in MSS CRC managed to match the overall *IFNG* (and associated signaling) output in MSI CRC. This brought into focus the origins and contexts of IFN-γ signaling. TCR repertoire analyses showed that, in fact, MSS CRC *IFNG*^+^CD8^+^ T cells had low TCR diversity and possessed largely viral TCRs. This was accompanied by heterogeneous inflammatory or tolerogenic features. Contrastingly, the MSI CRC *IFNG*^+^CD8^+^ T cells showed high TCR diversity and higher exclusive TCRs which associated with a higher neoantigen-reactive signature score. Accordingly, this was accompanied by higher tumor-reactive exhaustion and stem-like progenitor features as well as a TCR-activation signature. This emphasized that identical phenotypic orientations of CD8^+^ T cells may associate with completely different origins and context depending on the antigenic make-up of the tumors. This point was specifically captured on the level of *TOX*^HIGH^CD8^+^ T memory-exhausted cells, which showed different predicted TCR-specificities depending on CRC subtype. This highlighted how CD8^+^ T cells with TCRs predicted to bind non-tumor antigens in MSS CRC may resemble phenotypes of more tumor-relevant CD8^+^ T cells in MSI CRC, thus creating an ambiguous impression of ‘pseudo-hot’ MSS (MSI-like MSS)^54^. Hence, multi-dimensional deep dissection of CD8^+^ T cell transcriptomes, together with TCR repertoire, is needed to differentiate ‘pseudo-hot’ MSS from tumor-reactive ‘hot’ MSI tumors.

Although the TCR specificities were computationally predicted, the CD8^+^ T cell transcriptomes did associate with them in a manner consistent with published literature. For instance, MSI CRC CD8^+^ T cells with exclusive TCRs showed chronic exhaustion, and pro-immunogenic features. Such features associate with neoantigen-specific CD8^+^ T cells in high mutational burden tumors^48^. Similarly, tumors with low mutational burden have poor neoantigen-reactive CD8^+^ T cells. Accordingly, in MSS CRC, CD8^+^ T cells with exclusive TCRs showed less tumor-reactive exhaustion—an indication of heightened CD8^+^ T cell inactivation or absence of neoantigen-reactivity. Finally, terminal differentiation or long-term immunosurveillance characteristics have been connected to tumor irrelevant antigens^34,63,64,77^, a point substantiated in our data for TCRs predicted to bind self or viral-antigens. These transcriptomic results were validated on surface proteomic level with MSI CRC CD8^+^ T cells showing more effector memory phenotype than MSS CRC CD8^+^ T cells.

Apart from viral antigens, the gut microbiome can also be a dominant source of bystander activation^34,64^. Only in MSS CRC, the *IL17A*^+^ MAITs were particularly active and enriched for TCRs with predicted specificity against microbial riboflavin intermediates. A large diversity of microbes, including mycobacterium species can produce such intermediates^60,105^. The latter is interesting because, only in MSS CRC, we also observed mycobacterium infection-pathways as well as TCRs predicted to bind mycobacterium-derived epitopes in the *IFNG*^+^CD8^+^ T cell subset. This aligns with instances of mycobacterium infections in CRC patients^106^. It is likely that there are more bacterial epitope-specific TCRs in CRC, but these could not be captured due to their poor annotations in public databases. Finally, it still needs to be studied whether these microbial specificities are unique for MSS CRC or simply over-represented due to lower enrichment of tumor reactive CD8^+^ T cells.

Interestingly, we observed that the above tumor-reactive signaling modules could stratify MSI and MSS CRC TCGA patients, despite obvious imbalance in cellular resolution. Here, MSI CRC bifurcated into two clusters (ISC1a/1b) with highly contrasting immune landscapes and cohort-dependent prognostic differences. Contrastingly, MSS CRC bifurcated into two major clusters (ISC2/3; further composed of five subclusters i.e., ISC2a/2b/2c vs ISC3a/3b), which showed highly heterogeneous immune landscapes but with significant cohort-independent prognostic differences. We have summarized the key characteristics of the ISC system in Fig. 7f. More importantly, the ISC framework showed early potential as a machine learning-based score to differentiate ICB-immunotherapy responders from non-responders (at least in MSI CRC, with MSS CRC requiring more high-powered validation). This was also applicable at single cell resolution for signaling modules behind ISCs. Although promising, these results still require comprehensive validation in better powered cohorts applying immunotherapy in CRC patients. Indeed, a lack of sufficiently powered CRC patient cohort with immunotherapy interventions is a major limitation of our study. Beyond that more ex vivo functional T cell assays linked to TCR specificities of CRC patients need to be executed to validate our TCR repertoire conclusions.

Finally, our findings provide a preliminary explanation for why MSS CRC responds less favorably to PD-1 blockade but does respond to PD-1 and CTLA4 co-blockade. It is established that the efficacy of PD-1 blockade relies on pre-existing tumor-specific (neoantigen-reactive) TCRs^107^. However, this may not be the case for CTLA4 blockade, which induces diversification of TCR repertoire that is indiscriminate for its specificities. Hence, patients responding to CTLA4 blockade preferentially maintain high frequency of viral antigen-specific TCR clonotypes^107,108^. Such CD8^+^ T cells with tumor irrelevant specificities can induce antitumor immunity via at least two mechanisms: (I) they may respond to ICB-induced inflammation and elicit off-target antitumor activity via cytokines or cytolytic factors^109^ or (II) by possessing TCRs that cross-react with both viral/microbial antigens and tumor-relevant antigens^110,111^, thereby allowing initiation of collateral antigen-specific immunity. Interestingly, the latter type of cells show higher *EOMES* and *KLRG1* expression^110^, which were indeed associated with long-term immunosurveillance in our MSS CRC patients. Such possibilities require confirmation in a prospective clinical trial.

Our study provides a rich dataset of CD8^+^ T cell-states, TCR repertoire, transcriptional programs, and their immunotherapy relevance in a relatively large cohort of CRC patients. We provide a novel approach that organizes a heterogeneous set of transcriptional states and TCR specificities into ordered structures of mechanistically relevant subpopulations. Understanding the molecular mechanisms underlying these subpopulations and their association to immunotherapy response will be critical for advancing anti-CRC therapies^112^. Finally, our ISC classification system can guide the design of patient pre-selection strategies for applying innovative immunotherapy, especially for MSS CRC.

## Materials and methods

### CRC patient cohorts

For this study we have incorporated previously unpublished CRC patient samples (23 patients from SMC5) to complement the previously published datasets of CRC patients (40 patients in CRC16, KUL3, KUL5 and SMC) to be integrated in downstream analyses. We included data from all datasets^12^ previously published and approved by the local institutional review boards of Singhealth (2018–2795 and 2018–2376) for CRC-SG1 and CRC-SG2, Samsung Medical Center (approval no. SMC2017-07-131) for the SMC and Commissie Medische Ethiek UZ KU Leuven/Onderzoek (approval no. S50887-ML4707) for the KUL3 and KUL5 datasets, respectively. Of note, all research involving human participants, human material, and human data, was performed in accordance with the Declaration of Helsinki. Also, for all relevant human participants, informed consent to participate in the study was obtained in accordance with relevant institutional guidelines.

Survival information and other metadata features were updated for previous 21 SMC and SMC5 cohort patients according to the approved Institutional Review Board of Samsung Medical Center protocols (no. SMC 2017-07-131). Further details on the patient selection criteria for the patients in the other CRC16, SMC, KUL3 and KUL5 cohorts were published elsewhere^12,18^. All mentioned datasets/studies were conducted in accordance with ethical guidelines and all patients provided written, informed consent. A final list of included patients in this study can be found in Supplementary Table S1.

After resection, samples from both tumor and non-malignant colon tissues were collected and immediately transferred for tissue preparation. Tissues were subjected to single cell isolation, AllPrep DNA/RNA Mini Kit (QIAGEN) for DNA analysis and transcriptome sequencing.

### ScRNA-seq sample preparation

For the novel SMC5 samples, tissue dissociation was performed using a Tumor Dissociation Kit (Miltenyi Biotec) according to the manufacturer’s instructions. Briefly, tissues were cut into 2–4 mm-long pieces and transferred to C tubes containing an enzyme mix (enzymes H, R and A in Roswell Park Memorial Institute (RPMI) 1640 medium). Gentle MACS programs (h_tumor_01, 02 and 03) were run in a MACSmix Tube Rotator (Miltenyi) with two 30-min incubation periods at 37 °C between each run. The digested samples were filtered through a 70-µm strainer, purified using a Ficoll Paque PLUS (GE Healthcare) gradient and cryopreserved in CELLBANKER 1 (Zenoaq Pharma) before scRNA-seq. The cryopreserved single cell dissociates were rapidly thawed, washed and loaded into the Chromium system (10x Genomics) targeting 5000 cells. Following the manufacturer’s instructions, barcoded sequencing libraries were generated using Chromium Next GEM Single Cell 5′ v1.1 Reagent Kits and sequenced on a NovaSeq 6000 platform. Results were mapped to the GRCh38 human reference genome using CellRanger (10x Genomics, v3).

An additional enrichment of T cells was performed for two patients (SC040 and SC044) of the KUL5 dataset using and the REAlease® CD4/CD8 (TIL) MicroBead Kit (Miltenyi Biotec), prior to scRNA-seq similar to Joanito et al.^12^.

### CD8^**+**^ T cell single cell analysis

Single cell expression raw count data from CD8^+^ T cells from the five different single cell datasets (CRC16, KUL3, KUL5, SMC, SMC5) were stored in annotated data objects^113^ containing the raw count expression and the patient metadata table. These were further processed with Scanpy^114^ 1.8.1. Gene expression data were filtered to remove genes with zero count expression in all cells and non-protein coding genes. Mitochondrial and ribosomal genes were removed. Only cells with more than 200 genes expressed and more than 1000 reads per cell were retained. Single cell data for tumor MSI and MSS CRC cells were normalized using the “normalize_total” function from Scanpy^114^ with default parameters, except for “exclude_highly_variable=True” and log_2_-transformed. After reduction of technical variation with per-cell normalization, expression data for each scRNAseq dataset was batch corrected using pycombat^115^ to regress out technical variation attributed to the source dataset.

#### Comparison of batch correction methods

The previous batch-corrected dataset with Combat^115^ as described previously was used as reference. Subsequently, datasets were batch-corrected independently with representative examples of the various batch correction methods, such as ReCombat^116^, BBKNN^117^, Harmony^118^, Scanorama^119^, MNN_correct^120^, and Scanpy’s regress_out^114^. Gene expression for the genes defining the CD8^+^ T cell populations between the original Combat batch-corrected and the alternative batch-correction methods was compared as Spearman correlations. We opted to show the correlation in expression for 58 genes that are used for CD8^+^ T cell subtyping and immuno-regulation: *AHR, BATF, BCL6, BTLA, CCL3, CCL4, CCL5, CCR4, CCR5, CCR6, CCR7, CD27, CD28, CD4, CD44, PTPRC, CD8A, CD8B, CTLA4, CXCR3, CXCR4, CXCR5, CXCR6, ENTPD1, EOMES, FOXP3, GATA3, GZMA, GZMB, GZMK, ICOS, IFNG, IKZF2, IL10, IL21R, IL2RA, IL2RB, IL7R, ITGAE, KLF2, KLRB1, KLRG1, LAG3, MAF, PDCD1, PRF1, REL, SELL, STAT1, STAT3, STAT4, STAT6, TBX21, TIGIT, TNF, TNFRSF18, TNFRSF9, TOX*.

#### Comparison of joint vs independent batch correction

Datasets were batch corrected with Combat either joint (MSI and MSS) or separately. The agreement in full transcriptomics profiles differing only by computational correction was shown as a Spearman correlation. Spearman correlations were calculated with SciPy^121^ 1.11.1.

### Comprehensive CD8^+^ T cell map and subpopulation phenotypes

Following CD8^+^ T cell clustering for the CD8^+^ T cell populations for MSI and MSS CRC integrated datasets, dimensionality reduction (UMAP) was performed to obtain CD8^+^ T cell subpopulation clusters after Leiden clustering, 12 for MSI and 13 for MSS. Annotations of the conventional CD8^+^ T cells were performed using an expression-based scaling of gene expression signal within each population against previous knowledge of established markers of naive/progenitor, memory, exhaustion or effector/activation profiles (Supplementary Table S2). This was achieved at the level of gene expression visualization using Scanpy’s “dotplot” function with default parameters except for “standard_scale” = “var”. Similarly, for unconventional or invariant subpopulations we utilized *CD8A/B* expression instead as well as non-exclusive markers such as *LTA* and previously reported signatures such as Mucosal associated invariant T cells annotation that was further validated using MAIT specific signature expression quantification vs conventional CD8^+^ T cells^29^.

### *IFNG*^+^CD8^+^ T cells *IFNG* expression

Selection of *IFNG*^+^CD8^+^ T cells per patient was performed using a binarization approach of low expression values to high expression values using the average lowest expression between the two peaks (threshold 0.2) based on a density distribution of single cell expression of *IFNG* for each MSI, MSS, normal tissue adjacent to MSI tumor (NMSI) and normal tissue adjacent to MSS tumor (NMSS) dataset.

### Tumor-reactive signaling and NeoTCR signature quantifications

#### Identification of tumor-reactive signaling modules

Tumor-reactive signaling modules were identified based on co-expression correlation using distance matrices (Euclidean) and grouped by hierarchical distances of *IFNG*^+^CD8^+^ T cells, using the transcriptome.

#### Expression quantification of tumor-reactive signaling modules

Like the determination of IFNG^+^ cell state with thresholding, we calculated the activation map of the tumor-reactive signaling modules for each subpopulation annotation of *IFNG*^+^CD8^+^ T cell as average signal of binarized expression for MSI and MSS *IFNG*^+^CD8^+^ T cells respectively.

#### NeoTCR signature expression

The evaluation of signature expression for the NeoTCR8 signature^78^ was also computed as the mean of binarized expression per cell of NeoTCR signature genes (Supplementary Table S3) for *IFNG*^+^CD8^+^ T cells.

#### Tumor-reactive signaling module overlap

For each of the 39 genes defining the tumor-reactive signaling modules, presence, or absence of the gene in the signature was marked with a black square (presence) or white (absence). Overlaps were visualized with seaborn^122^ 0.11.1 and matplotlib^123^ 3.5.1 and clustered using the Jaccard similarity index.

#### Overrepresentation analysis of tumor-reactive signatures

The 39 genes were submitted to Webgestalt^41^ on 28/05/2023 and subjected to the standard overrepresentation analysis in the Reactome pathway database, for which we selected the protein-coding genome as reference background. All other default settings were maintained.

### TCR activation and bystander activation signaling

TCR activation signaling and bystander activation signaling for *IFNG*^+^CD8^+^ T cells were calculated on normal MSI, tumor MSI, normal MSS and tumor MSS cells independently. Signature quantification was performed on CD4-derived signatures from a study^65^ describing TCR*-*associated activation and bystander activation based on differential expression (resting vs activated) that was published on the MSIGDB^124^ v.7.2 (GMT’s c7 all, GSE13738). The TCR activation signal and the bystander activation signal were then calculated as the Spearman correlation values to the average expression of the published expression data from GSE13738 corresponding to experimental TCR and Bystander activation. The final signal represents the Spearman correlation score for each cell and for each TCR activation and bystander activation gene expression signature. The difference in these correlation scores was used as a measure of directionality between TCR activation and bystander activation score. Finally, after defining TCR and Bystander-activation high and low, four quadrants were defined crossing high and low areas to perform a Welch test statistical cell quantification difference between MSI and MSS tumor quadrants.

#### DE of TCR activation^HI^ cells

Differential expression of single cell genes was then performed between quadrants to identify gene expression changes to the TCR^LOW^Bystander^LOW^ quadrant population using Scanpy’s “rank_genes_groups” function. Top DE genes filtered by *P* value < 0.05 in the TCR activation comparison as well as log_2_FC > 0 were further selected for a Reactome pathway analysis using Cytoscape 3.8.2 with the ClueGo^24^ 2.5.8 plug-in. Reactome pathways were grouped by pathway gene overlaps and gene networks extended to show all genes included in the analysis as well as important genes for each pathway.

### Single-cell VDJ sequencing

For both KUL5 and SMC5, V(D)J enriched libraries were sequenced on an Illumina HiSeq4000 and TCR alignment and annotation was achieved with CellRanger VDJ (10x Genomics).

### ScTCR sequencing analysis

Raw TCR sequencing data from 12 CRC MSS and 16 CRC MSI patients were loaded into Scirpy^70^ 0.10.1 for a TCR analysis. Scirpy is an extension of Scanpy for analyzing single cell immune-cell receptor sequencing data (Pandas 1.3.3, Scanpy 1.8.1, Anndata 0.7.6, SciPy 1.5.1, Seaborn 0.10.1, Matplotlib 3.5.1, Statannot 0.2.3).

TCR relevant information, such as TCR type, CDR3 nucleotide sequences, CDR3 amino acid sequences, CDR3 read counts, VDJ genes and chain productivity were extracted and transformed into AirrCell objects, which represent the community standard AIRR rearrangement schema. Transformation into this format was performed based on the Scirpy documentation. AirrCell objects were subsequently transformed into an annotated data object with the “scirpy.from_airr_cells” function built into Scirpy. Sample ID, tissue, MSI status, patient ID, cell annotation, cell annotation subtype, IFNG status and bystander status were added as observation data to the AnnData object.

To search for potential matches with already known TCR–antigen interactions, the CDR3 sequences were queried against the epitope databases IEDB^71^. IEDB epitope data were externally retrieved from the IEDB website under (https://www.iedb.org/database_export_v3.php), contained in the file “receptor_full_v3.zip”. Downloaded IEDB data was turned into AirrCell objects and then to AnnData objects in the same manner as the CRC TCR. The AnnData data contain the database’s specific antigen information; IEDB reports the antigen organism, as well as info on the specific antigen.

After the IEDB AnnData objects were created, annotation of TCRs was performed with Scirpy’s “scirpy.tl.ir_query_annotate” function, using the “alignment” metric, so certain mismatches were allowed. We used the “most-frequent” strategy, where the most frequent match is reported. If a tie occurs, the TCR preference is labeled as “ambiguous”. If no match was found, the antigen was labeled as “Unknown”. To make comparisons and visualization easier, matched IEDB antigen organisms were divided into several major groups: viral, bacterial, self, exclusive, shared, and unknown. For further steps, the annotated TCRs were split into two groups: patients with MSI status and patients with MSS status. All the following procedures were performed for MSI and MSS individually.

Next, the clonotypes and clonotype clusters were defined. Clonotypes are a collection of cells that bear the same adaptive immune receptors and therefore recognize the same epitopes, while clonotype clusters connect clonotypes having similar immune receptors. First, “scripy.pp.ir_dist” was used to calculate the distances between CDR3 nucleotide or amino acid sequences, based on sequence identity for clonotypes, or similarity for clonotype clusters, respectively. We calculated similarity between clonotypes, using pairwise sequence alignment as part of the Biopython^125^ library with the distance defined by the BLOSUM62 substitution matrix. For the similarity calculation, the options, metric = ”alignment”, sequence = ”aa” were added and a score cut-off = 15 was set. Then we defined the clonotypes using “scirpy.tl.define_clonotypes”.

The Scirpy function “scirpy.tl.clonotype_network” is used to compute the network layout. All cells with a distance between their CDR3 sequences lower than the cut-off will be connected to clonotype clusters. Visualization is achieved with “scirpy.pl.clonotype_network”, clonotypes are colored by the various observation columns, e.g., “color = Organism” or “color = MSI”. The alpha diversity of clonotypes within antigen organisms from IEDB, as well as within the groups (viral, bacterial, exclusive, shared cancer, self) was computed with the function “scirpy.tl.alpha_diversity”, using the default “normalized_shannon_entropy” as metric. Also, the richness and evenness were calculated within organism and combined groups, providing scikit-bio’s “chao1” as metric for richness and “heip_e” for evenness (skbio.diversity.alpha). A table matching epitopes and epitope targets is provided in the supplementary materials (Supplementary Table S5).

#### TCR groups DE and GSEA

Differential expression for cells within each antigen epitope group was performed using Scanpy’s “rank_genes_groups” using logistic regression method with additional parameters such as penalty = “elasticnet’, l1_ratio = 0, max_iter = 1000, solver = “saga”, corr_method = “bonferroni”. Pathway enrichments were calculated with Gseapy^126^ 1.0.4, using the Reactome terms included in the MSIGDB v.7.2 release, using the pre-ranked method using the Scanpy scores as ranking criterion. 2000 iterations were used for FDR estimation.

#### Exclusive TCR map to normal adjacent TCRs

Exclusive TCR mapping between normal adjacent tissue and MSI and MSS CRC TCRs was performed on beta chains map score using their amino acid aa sequences as local alignment identity in percentage to the query aa sequences of tumor cells. For that we first calculated the maximum alignment score for each tumor sequence using “pairwise2” from Biopython, using Blosum62 as substitution matrix. Default parameters were used, except for op = −4 and ep = −4. The final query TCR sequence score was calculated as a proportion to the tumor TCR maximum score for each of the tumor TCR sequences against the normal adjacent tissue TCR sequences using the same AA local alignment parameters. Similar or different TCR sequences were quantified with a percentage-based pairwise alignment score threshold of 80% identity.

### Single-cell MAIT TCR sequencing analysis

Sequences of MAIT TCR alpha and beta chains were scored using their amino acid sequences as local alignment identity (%) to the known aa sequences of MAIT cells^61^. To this end, we first calculated the maximum alignment score for each known sequence using “pairwise2” from Biopython with the substitution matrix Blosum62 and default parameters, except for op = –4 and ep = –4. The final query TCR sequence score was calculated as a proportion to the known maximum score for each of the known MAIT TCR sequences against the query TCR sequence using the same AA local alignment parameters as the known TCR sequences. Finally, we multiplied the identity score results for the alpha and beta chains for each cell and selected only the highest score as final representation of maximum possible MAIT TCR aa sequence alignment for each cell.

### Multiple factor annotation analysis

The overlap analysis of multiple layers of per cell annotation as factors was performed using the package FactoMineR^127^ in R. The four layers of annotation integrated in the form of a multiple correspondence analysis including the binarized *IFNG* expression (see methods), the CD8^+^ T cell subpopulations, the TCR predicted epitope groups and the binarized tumor-reactive signaling modules. The later was performed dividing the score per cell for each signaling modules between <= 0.5 or > 0.5.

### CyTOF analysis

Datasets were downloaded from FlowRepository accession FR-FCM-Z24H^80^ and processed with Pytometry 0.1.3 (BioRXiv, 10.1101/2022.10.10.511546). Values were arcsinh transformed with cofactor 5. Only live cells were retained and further selected for positivity of CD3 and CD8. Subsequently, cells expressing CD4, CD86, CD115 or CD163 were filtered out. For the remaining cells, UMAPs were calculated using Scanpy 1.9.3, with the neighbors defined by the diffusion maps from Palantir^128^ 1.2, using random seed 770 and 30 neighbors and calculated with Scanpy’s “sc.pp.neighbors”. Leiden clustering was performed with resolution 0.03. Trajectories were inferred with scFates^129^, using principal tree inference settings that specified 200 nodes, random seed 1, ppt_lambda as 200, ppt_sigma as 0.2 and ppt_nsteps defined as 100. Pseudotime was inferred with n_map 100 and random seed 770. Node 60 was defined as the root. Values for several proteomics markers were overlaid on the trajectory using scFates’ “scf.pl.trajectory”.

### Stratification of MSI and MSS CRC patients based on scRNA-seq signaling modules

The tumor-reactive signaling modules as binarized genetic signatures for each single cell were used to stratify the patients depending on their signaling activation by using a hierarchical clustering of per patient mean values of each tumor-reactive signaling module on *IFNG*^+^CD8^+^ T cells. These patient averages for MSI CRC patients were stratified into two clusters, whereas MSS CRC patients were stratified into five clusters by using a hierarchical clustering approach, visualized with Seaborn’s “clustermap” function with method = “complete” and metric = “Euclidean”.

### Stratification of bulk primary tumor transcriptomes for MSI and MSS CRC patients

#### CRC immune subclassification

Single *IFNG*^+^CD8^+^ T cells and their derived subpopulation phenotypes were used to define select genes (e.g., IFNG, LAG3, JAK3) associated with each of these phenotypes. Due to the prior decision based on differences in disease type, MSI and MSS were treated separately. For each phenotype signature, pairwise distances between patients were calculated as the Spearman correlation over the vector defined by the expression of the genes in this signature. Distances between patients were then averaged over the signatures, to obtain a two-dimensional space, in which agglomerative clustering was used to define the number of groups, with n maximally set as 5 groups. Datasets were processed with Pandas 1.2.4 and SciPy 1.6.3. Clustering was performed with scikit-learn^130^ 0.24.1. Resulting patient-to-patient distances and corresponding ISC labels are provided as supplementary material (Supplementary Table S6) and can be used as templates to train machine learning classifiers for the ISC classification system.

#### Cell deconvolutions and signature quantifications of TCGA patients

The signal quantification of such signaling modules was calculated for each ISC cluster previously identified for MSI and MSS patient samples and normalized across all TCGA ISC clusters including immune cell quantification (QUANTISEQ immune-deconvolution)^131^, antigenicity parameters (Indel/SNV neoantigens, TCR/BCR Richness)^73^, immune-landscape signatures^73^ and H&E tumor image-based deep learning results^92^ for tumor-infiltrating lymphocytes (TILs). Immune deconvolutions for TCGA were obtained from TIMER 2.0 (http://timer.cistrome.org). H&E-derived observations^92^ and processed data from the Immune landscape of cancer were downloaded from their corresponding GDC webpages (https://gdc.cancer.gov/). Results were averaged using the arithmetic mean and compared with Welch *t*-tests (*P* < 0.05) using SciPy 1.6.2.

#### TCR activation and bystander activation of TCGA patients

TCR activation signaling to bystander activation signaling ratio for each of the ISC clusters from TCGA MSI and MSS patient data was calculated as the ratio of the metagenes (average gene expression in the signature) for the CD4 TCR activation and bystander activation signatures per patient. These were then statistically compared per ISC category using a Welch two-sample *t*-test (*P* < 0.05) with SciPy 1.6.2.

### Machine learning for ISC label prediction on GTex and Keynote-177

Clinical trial information for MSI (Keynote-177)^17^ was obtained from the original authors per request. Gene expression data from TCGA were obtained from the Toil-recompute hub on Xena^132^. Per trial, datasets were L1-normalized and collated to the L1-normalized TCGA MSI or MSS dataset depending on the respective situation. Patients listed as MSI-H in the TCGA classification were allocated to the MSI subtype. MSI-L and MSS TCGA labels were added to MSS, as evidence for the former’s state as MSI is contested. Batch correction was performed using pyCombat. Models were trained and tested using a repeated fivefold cross-validation approach (random forest wrapper^133^) to derive top-ranking genes associated with ISC label prediction. Top ranks were determined using the averaged feature importance (mean impurity decrease) in the random forest classification models, which was essential to prevent overfitting. Using the most important genes (threshold: score > 0.001), a new model was trained on the full MSI or MSS TCGA training set using only these genes and then applied to the clinical trial dataset. Immune deconvolutions for the clinical trials were calculated with TIMER 2.0. CMS subtypes were calculated with CMSCaller^134^ 0.99.2 in R 4.1.1. Statistical significance was evaluated using the Welch two-sample *t*-test implemented in SciPy 1.6.2, with significance threshold of 0.05. The random forest classification model was based on the implementation in scikit-learn 0.24.1. Sankey diagrams linking ISC to CMS labels were created with Plotly 5.14.1.

For the TCGA to Gtex comparison, samples were batch-corrected with Combat using only the normal TCGA samples. This transformation matrix was afterwards also applied to the TCGA tumor samples for the UMAP representation. We then applied the TCGA ISC Random Forest classifier (random_state = 12, n_estimators = 5000, class_weight = balanced) to the TCGA and Gtex normal samples. Normal sample calls and tumor samples were present in a single UMAP with UMAP-learn 0.5.3 using all genes and seed 770. For Keynote-177, only TCGA MSI-H samples were used and batch-corrected with the Keynote-177 MSI patient samples.

### Survival analysis for TCGA and SIDRA-LUMC

TCGA expression data and survival information were obtained from Xena. Survival data for the SIDRA-LUMC cohort^101^, as well the expression profiles were provided with the referenced study. For both cohorts, progression-free survival information was used (TCGA: progression-free interval, PFI; SIDRA-LUMC: progression-free survival, PFS). Survival statistics were computed with lifelines 0.27.4 using Kaplan–Meyer curves using default settings. All patient samples were used for survival information was available. Patients were aggregated in inflammatory (ISC2) and non-inflammatory/desert-like (ISC3) subtypes. Statistical significance in survival between these two groups was tested using the log rank test, with *P* < 0.05 as significance threshold. Multivariate analyses (CoxPH) were also performed for both TCGA (correcting for age, gender, and tumor purity), as well as SIDRA-LUMC (correcting for age and gender). All samples for which these metrics were available were used. CMS and MSI calls were used as provided by the original authors. The Cox Proportional Hazards models were created using lifelines 0.27.4.

### Microbiome predictions

Microbiome counts were downloaded from the FTP site associated with the Poore et al. Nature study^95^, in which thousands of viral and bacterial genomes were aligned to a database with Kraken. In the CRC TCGA patient cohort, 84% of reads were viral, 9% viral and 7% had a fungal origin. An XGBoost^135^ machine learning classifier was trained with scikit-learn 1.2.2 and XGBoost 1.7.6. The objective function was set as binary:logistic. Hyperparameters were tuned using a grid search for balanced accuracy, which ended up selecting n_estimaters as 75. Other settings were kept as default, other than the random seed, which was defined as 770. Classifier validity was tested with 5-fold cross-validation. The calculated metrics were precision, recall, F1-score, and Matthews correlation coefficient. ROC curves were plotted using the function “RocCurveDisplay.from_predictions”. Corresponding Shapley feature importance values from the final model were obtained using Shapley values calculated with Shap 0.42.1.

### Single-cell MSI analysis for tumor-reactive landscape components

A single cell dataset^104^ containing 19 MSI patients (GSE205506) was downloaded from the Gene Expression Omnibus and processed with BioTuring 3. Z-scores representing normalized gene expression in the CD8^+^ T cell compartment were aggregated to create metagenes (average normalized expression for all genes in set) for the tumor-reactive signaling modules. Signature expression scores split per treatment category (untreated, anti-PD-1, anti-PD-1+celecoxib) or per response group (pCR, non-pCR) were represented as boxplots using Seaborn 0.12.2 and Matplotlib 3.7.1. UMAPs representing patient, treatment or response distribution were created based on signaling module expression with UMAP-learn 0.5.3 (seed = 770).

### Supplementary information

Supplementary Figures and legends

Supplementary Table S1

Supplementary Table S2

Supplementary Table S3

Supplementary Table S4

Supplementary Table S5

Supplementary Table S6

## Data Availability

All major datasets (and associated meta-data and/or codes) analyzed for this project are either available on Synapse (Syn ID: syn41687327) or within the Supplementary Tables/Materials accompanying this manuscript. Other publicly available datasets used are available elsewhere such as TCGA data, publicly available from Xena – (http://xena.ucsc.edu), FireBrowse portal (a Broad Institute GDAC Firehose analyses pipeline: http://firebrowse.org/), or TCGA PanCancerAtlas Immune Response Working Group’s Cancer Research Institute (CRI) iAtlas Explorer (https://gdc.cancer.gov/about-data/publications/panimmune). For references to studies from where specific omics datasets were accessed, please see the suitable citations in-text or in methods sections.

## Code availability

All major datasets (and associated meta-data and/or codes) analyzed for this project are available on Synapse (Syn ID: syn41687327). Other publicly available datasets used are available elsewhere such as TCGA data, publicly available from Xena – (http://xena.ucsc.edu), FireBrowse portal (a Broad Institute GDAC Firehose analyses pipeline: http://firebrowse.org/), or TCGA PanCancerAtlas Immune Response Working Group’s Cancer Research Institute (CRI) iAtlas Explorer (https://gdc.cancer.gov/about-data/publications/panimmune). For references to studies from where specific omics datasets were accessed, please see the suitable citations in-text or in methods sections.
