# Supplementary material for: Single cell dynamics of tumor specificity vs bystander activity in CD8+ T cells define the diverse immune landscapes in colorectal cancer
Source: Cell Discov. 2023 Nov 15;9:114. doi: 10.1038/s41421-023-00605-4 (PMC10652011; doi:10.1038/s41421-023-00605-4)
Supplement: Supplementary file 1 — Supplementary Figures and legends [file 41421_2023_605_MOESM1_ESM.pdf]

SUPPLEMENTARY FIGURES & LEGENDS

Single cell dynamics of tumour specificity vs. bystander activity in $CD8^{+}$T cells define the diverse immune landscapes in colorectal cancer

Correspondence: abhishek.garg@kuleuven.be or sabine.tejpar@kuleuven.be

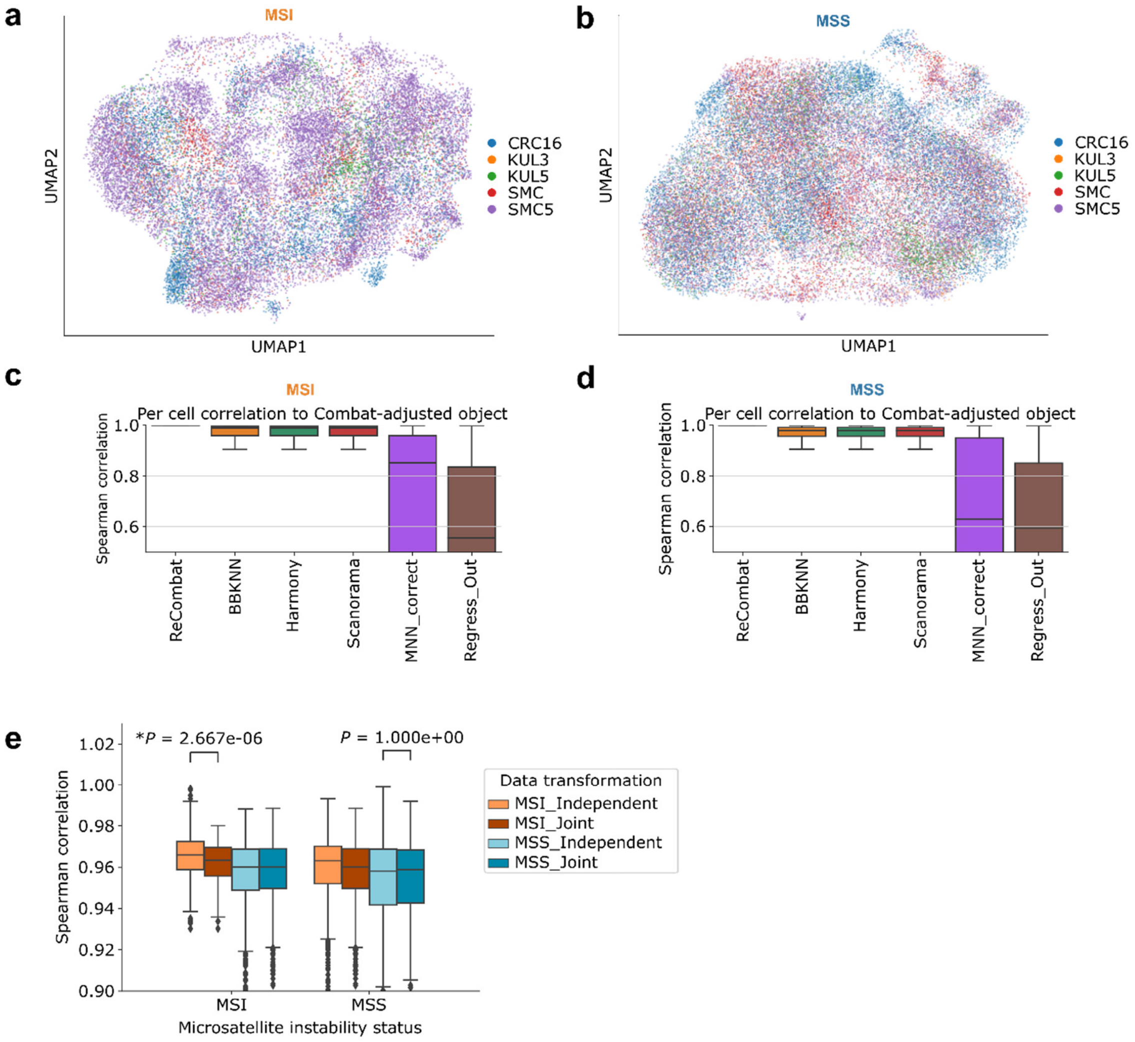

Supplementary Figure 1. Batch correcting the single-cell datasets. (a-b) UMAP representations of the 11420 MSI CD8$^+$T cells across 22 MSI CRC patients **(a)** and 17353 MSS CD8$^+$ T cells in 41 MSS CRC patients **(b)** in the five single-cell datasets (CRC16, KUL3, KUL5, SMC, SMC5) after quality control with Scanpy and subsequent batch correction with Combat. **(c-d)** Boxplots showing Spearman correlation between Combat batch correction and a selection of batch correction alternatives commonly used in single-cell analysis for the set of genes that was used for annotating the distinct CD8$^+$ T cell populations in MSI **(c)** and MSS **(d)**. **(e)** Spearman correlation between full single-cell transcriptomes prior to and after batch correction with Combat when MSI and MSS are processed individually, as well as jointly.

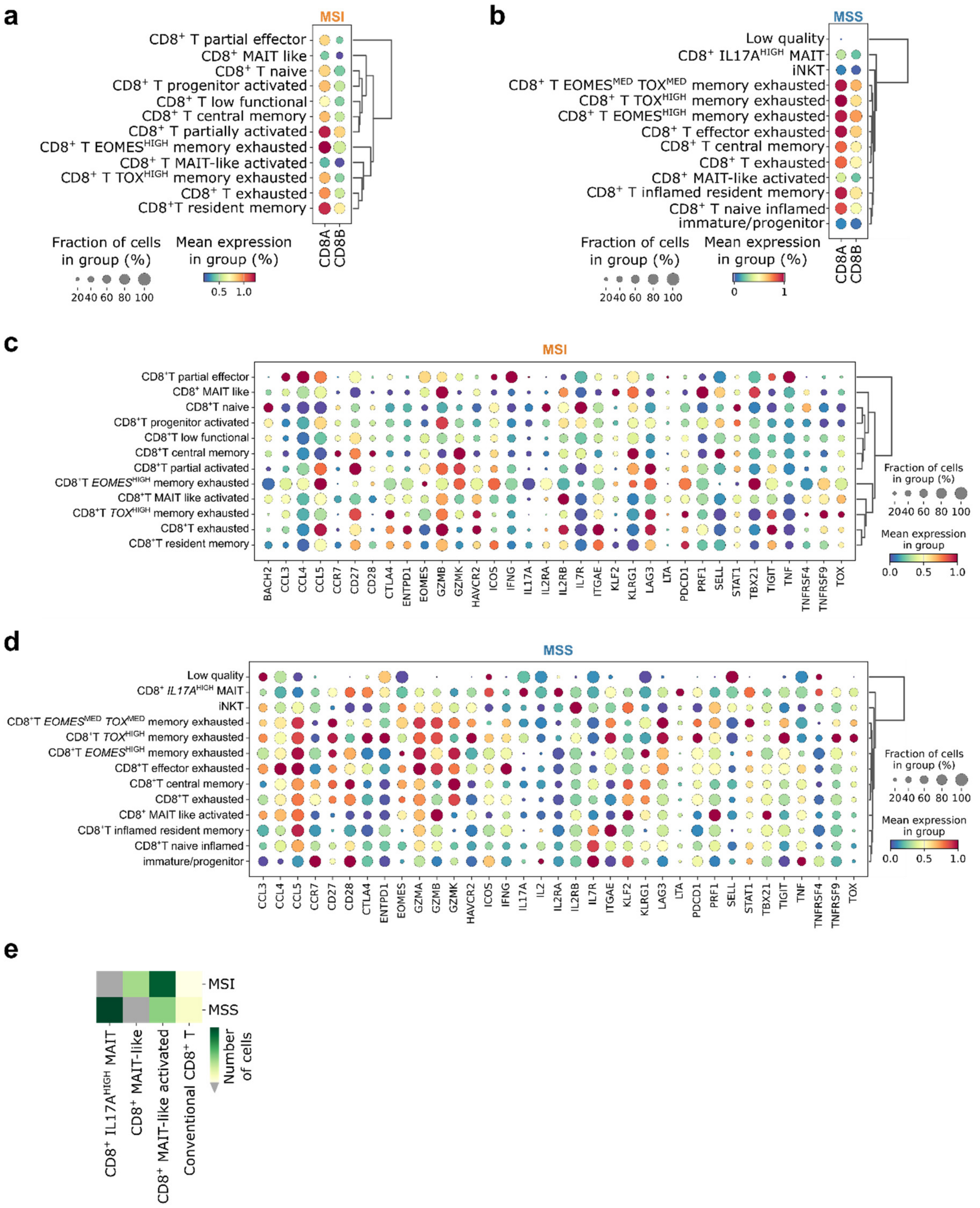

Supplementary Figure 2. Annotation of the CD8$^+$ T cell subtypes. (a-b) Expression of the CD8A and CD8B genes in MSI **(a)** and MSS **(b)**. **(c-d)** Dot plots illustrating expression of well-known literature marker genes for CD8$^+$ T cell subpopulation annotation for MSI **(c)** and MSS **(d)**. The markers for conventional CD8$^+$T cells, naïve/progenitor, memory, exhaustion or effector/activation markers of CD8$^+$ T cells are shown. **(e)** Comparison of MAIT and MAIT-like

T cell population sizes between MSI (1486 cells) and MSS (2540 cells), expressing MAIT-specific signature expression versus conventional $CD8^+$ T cells.

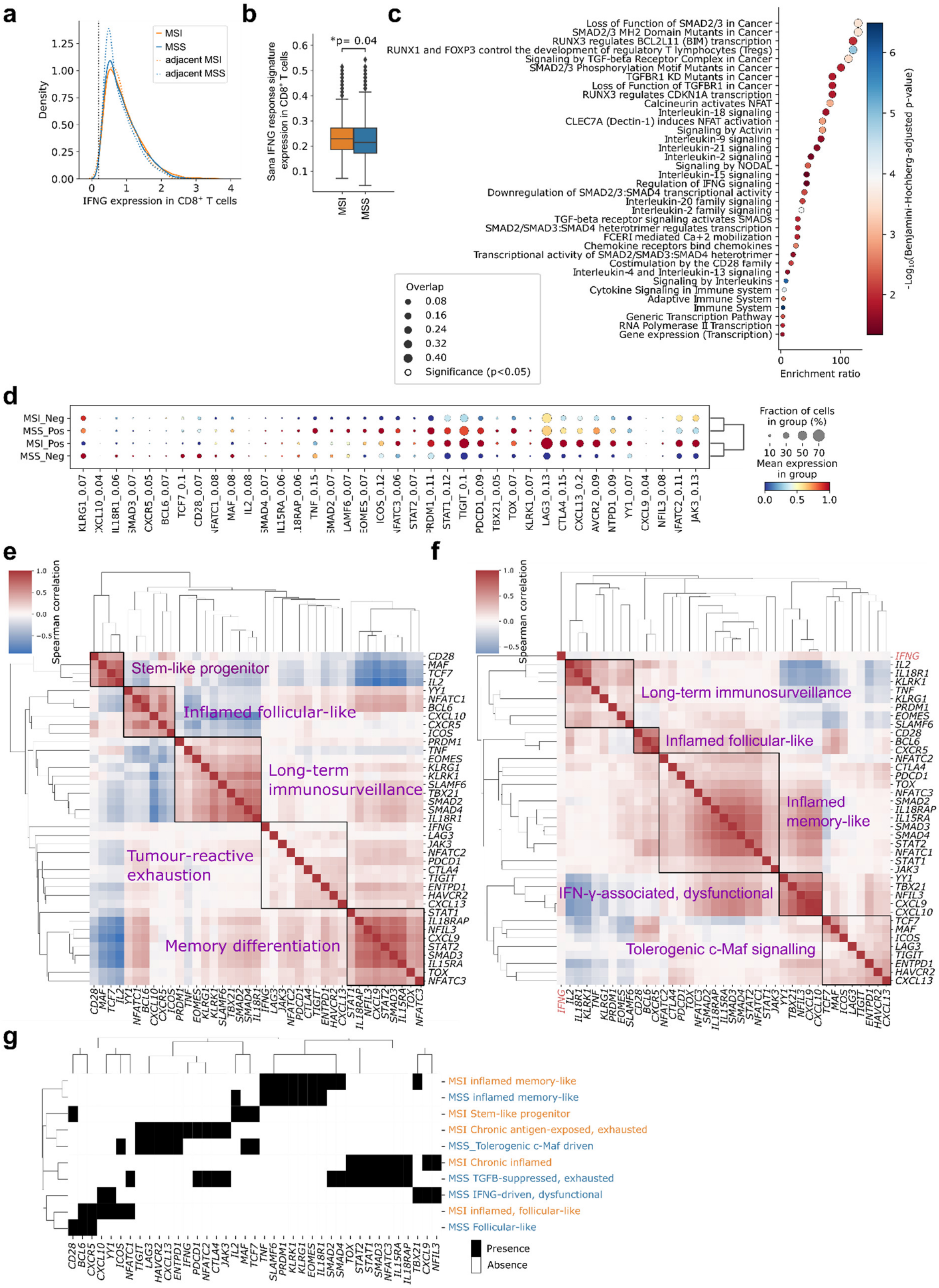
a
b
c
d
e
f
g
MSI
MSS
adjacent MSI
adjacent MSS
Density
IFNG expression in CD8⁺ T cells
*p= 0.04
Sana IFNG response signature expression in CD8⁺ T cells
MSI
MSS
Loss of Function of SMAD2/3 in Cancer
SMAD2/3 MH2 Domain Mutants in Cancer
RUNX3 regulates BCL2L11 (BIM) transcription
RUNX1 and FOXP3 control the development of regulatory T lymphocytes (Tregs)
Signaling by TGF-beta Receptor Complex in Cancer
SMAD2/3 Phosphorylation Motif Mutants in Cancer
TGFBR1 KD Mutants in Cancer
Loss of Function of TGFBR1 in Cancer
RUNX3 regulates CDKN1A transcription
Calcineurin activates NFAT
Interleukin-18 signaling
CLEC7A (Dectin-1) induces NFAT activation
Signaling by Activin
Interleukin-9 signaling
Interleukin-21 signaling
Interleukin-2 signaling
Signaling by NODAL
Interleukin-15 signaling
Regulation of IFNG signaling
Downregulation of SMAD2/3:SMAD4 transcriptional activity
Interleukin-20 family signaling
Interleukin-2 family signaling
TGF-beta receptor signaling activates SMADs
SMAD2/SMAD3:SMAD4 heterotrimer regulates transcription
FCERI mediated Ca+2 mobilization
Chemokine receptors bind chemokines
Transcriptional activity of SMAD2/SMAD3:SMAD4 heterotrimer
Costimulation by the CD28 family
Interleukin-4 and Interleukin-13 signaling
Signaling by Interleukins
Cytokine Signaling in Immune system
Adaptive Immune System
Immune System
Generic Transcription Pathway
RNA Polymerase II Transcription
Gene expression (Transcription)
Overlap
Significance (p<0.05)
Enrichment ratio
-Log10(Benjamini-Hochberg-adjusted p-value)
MSI_Neg
MSS_Pos
MSI_Pos
MSS_Neg
Fraction of cells in group (%)
Mean expression in group
Spearman correlation
Stem-like progenitor
Inflamed follicular-like
Long-term immunosurveillance
Tumour-reactive exhaustion
Memory differentiation
Inflamed memory-like
IFN-γ-associated, dysfunctional
Tolerogenic c-Maf signalling
MSI inflamed memory-like
MSS inflamed memory-like
MSI Stem-like progenitor
MSI Chronic antigen-exposed, exhausted
MSS_Tolerogenic c-Maf driven
MSI Chronic inflamed
MSS TGFB-suppressed, exhausted
MSS IFNG-driven, dysfunctional
MSI inflamed, follicular-like
MSS Follicular-like
Presence
Absence

Supplementary Figure 3. IFNG and tumour-reactive signalling modules. (a) Overall density distribution of IFNG expression for all $CD8^+$ T cells in MSI tumour (orange, 11420 cells), normal tissue adjacent to MSI tumours (dashed orange, 1958 cells), MSS tumour (blue, 17353 cells) and normal tissue adjacent to MSS tumours (dashed blue, 7867 cells). **(b)** Boxplot of an *IFNG* response signature from MSigDB (SANA_RESPONSE_TO_IFNG_UP,) as average of scaled gene expression. Statistical testing between groups was performed using the two-sample t-test for independent samples with p-value threshold <0.05. **(c)** Overrepresentation analysis using Reactome terms on the gene set of 39 genes that defines five tumour-reactive modules for $CD8^+$ T cells in MSI and five in MSS CRC patients. **(d)** Dot plot of gene expression of the 39 tumour-reactive signature genes in MSI and MSS, divided into IFNG positive (Pos) and negative (Neg) $CD8^+$ T cells. **(e-f)** Correlation matrices showing Spearman correlations between the 39 tumour-reactive module-defining genes in MSI **(e)** and MSS **(f)** Corresponding label annotations are shown in purple. **(g)** Composition of the tumour-reactive gene signatures. Presence of a gene in a module signature is indicated with a black box.

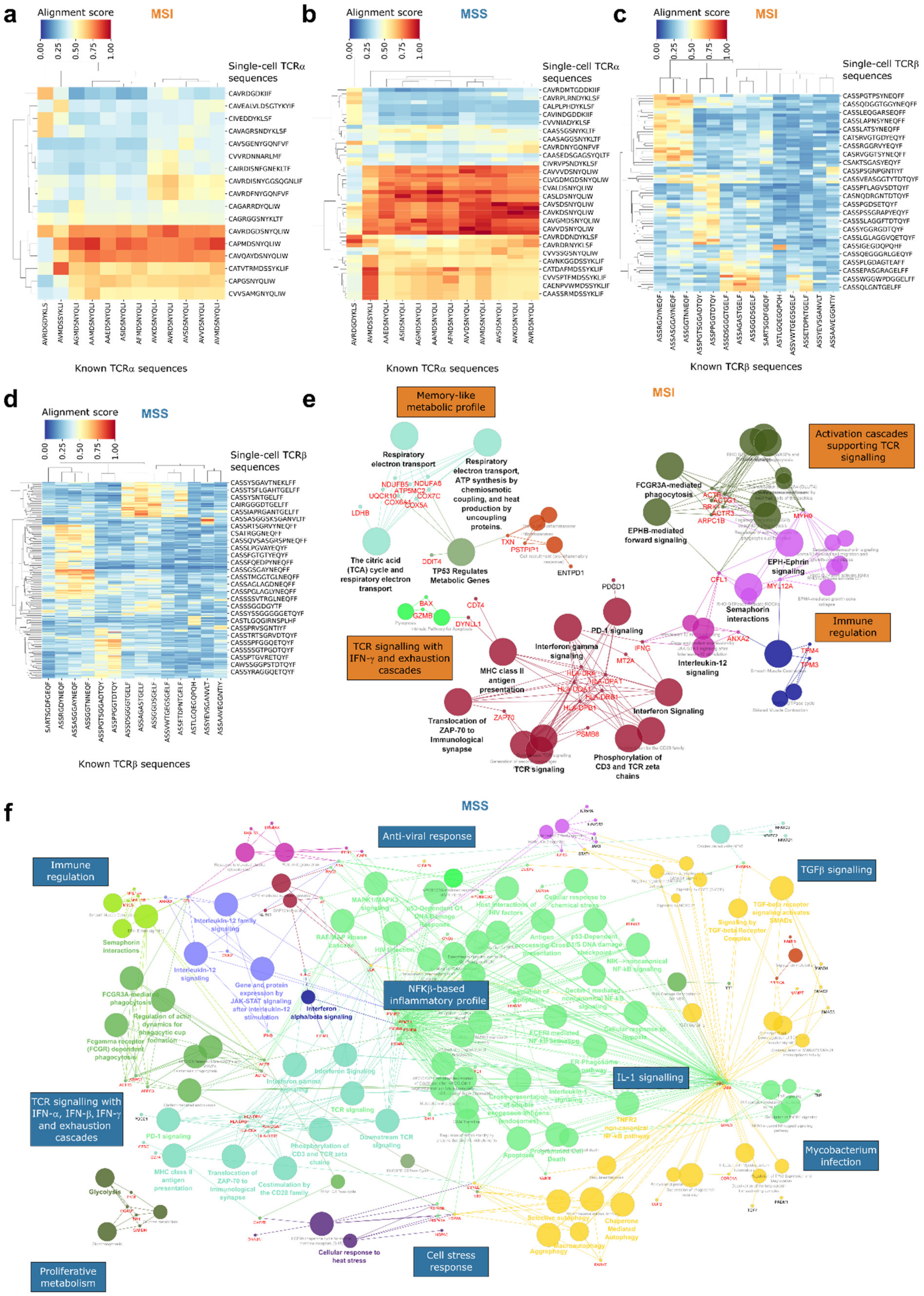

a
Alignment score
MSI
0.00
0.25
0.50
0.75
1.00
Single-cell TCRα sequences
Known TCRα sequences
b
Alignment score
MSS
Single-cell TCRα sequences
Known TCRα sequences
c
Alignment score
MSI
Single-cell TCRβ sequences
Known TCRβ sequences
d
Alignment score
MSS
Single-cell TCRβ sequences
Known TCRβ sequences
e
MSI
Memory-like metabolic profile
Activation cascades supporting TCR signalling
TCR signalling with IFN-γ and exhaustion cascades
Immune regulation
Respiratory electron transport
Respiratory electron transport, ATP synthesis by chemiosmotic coupling, and heat production by uncoupling proteins.
The citric acid (TCA) cycle and respiratory electron transport
TP53 Regulates Metabolic Genes
FCGR3A-mediated phagocytosis
EPHB-mediated forward signaling
EPH-Ephrin signaling
Semaphorin interactions
PD-1 signaling
Interferon gamma signaling
Interleukin-12 signaling
MHC class II antigen presentation
Interferon Signaling
Translocation of ZAP-70 to Immunological synapse
TCR signaling
Phosphorylation of CD3 and TCR zeta chains
f
MSS
Anti-viral response
Immune regulation
TGFβ signalling
NFKβ-based inflammatory profile
IL-1 signalling
TCR signalling with IFN-α, IFN-β, IFN-γ and exhaustion cascades
Mycobacterium infection
Cell stress response
Proliferative metabolism

Supplementary Figure 4. MAIT TCR alignment and graph-based representation of pathway enrichment in TCR activation and bystander activation signatures. (a-d) Pairwise TCR sequence alignment using the BLOSUM62 substitution matrix between known MAIT TCR sequences and MAIT TCR sequences obtained from the single-cell TCR-seq. Pairwise alignments were performed. Calculations were based on alignment-based identity scores of alpha **(a-b)** and beta chains **(c-d)** for MSI **(a, c)** and MSS **(b, d)**. **(e-f)** Graph representation of pathway enrichment analysis with Reactome terms using differentially expressed genes from the TCR activation and bystander activation signatures in MSI **(e)** and MSS **(f)**. Pathways were grouped by pathway gene overlap (colours). The networks were extended to show all connected genes for each enriched pathway (red).

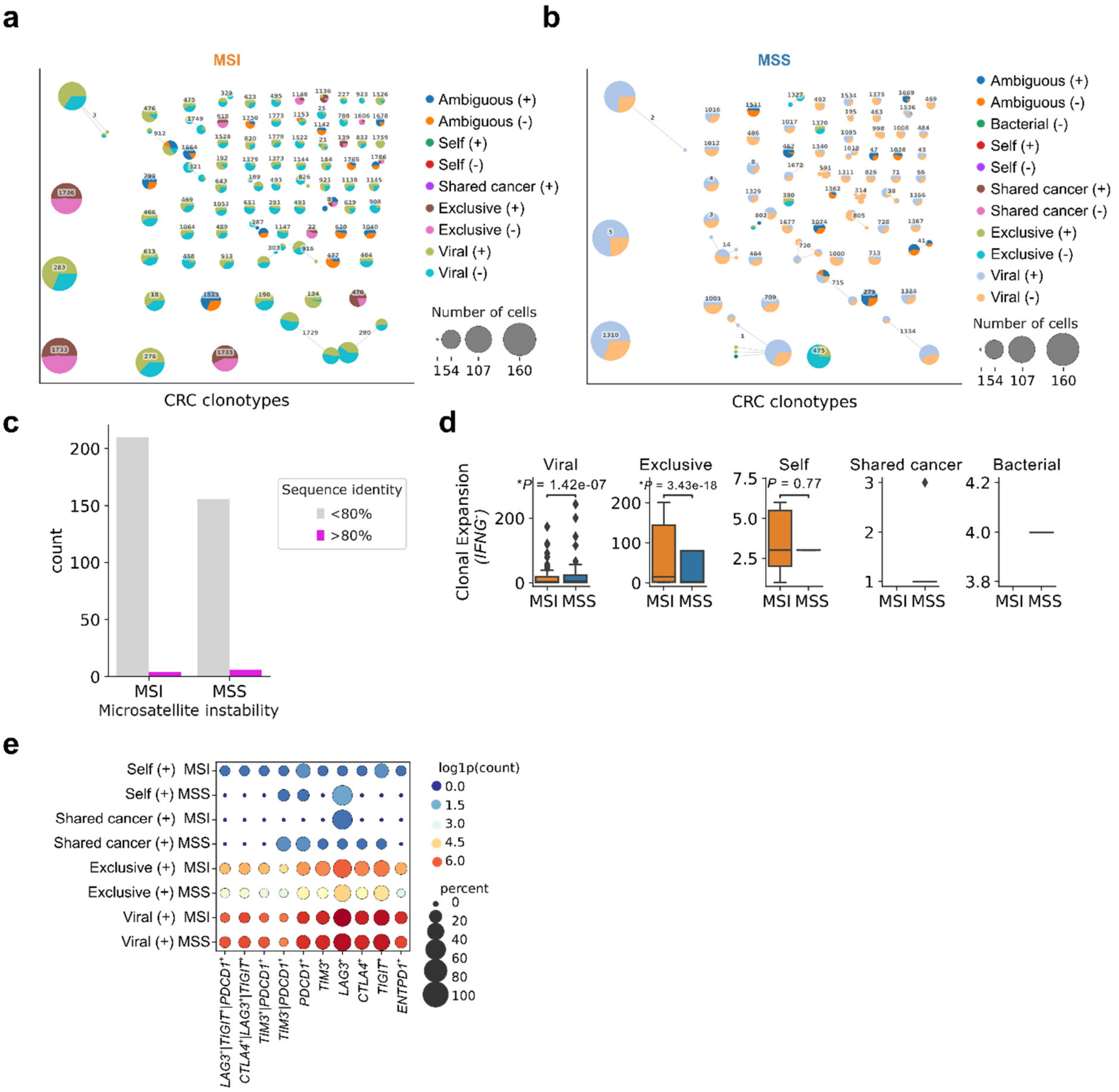

Supplementary Figure 5. Results from single cell TCR sequencing. (a-b) Clonotype identification and annotation of the antigen epitopes targeted by the TCR sequences for MSI **(a**, 1896 unique clones**)** and MSS **(b**, 1871 unique clones**)** samples. **(c)** Indication of TCR sequence identity overlap between tumour and normal adjacent tissue associated TCR sequences i.e., above 80% (purple) or below (grey) (y axis) in both CRC subtypes. **(d)** Boxplots illustrating clonal expansion (as number of cells per clonotype) for each TCR target epitope group in IFNG^{-} CD8^{+} T cells (viral: 3219, exclusive: 649, self: 16, shared: 5, bacterial: 4) in MSI and MSS. **(e)** Dot plot illustrating the number of IFNG^{+} CD8^{+} T cells with gene expression exceeding the threshold values for a range of known immune checkpoints that are currently being evaluated in clinical trials. Cells were grouped by microsatellite subtype status and TCR target epitope preference.

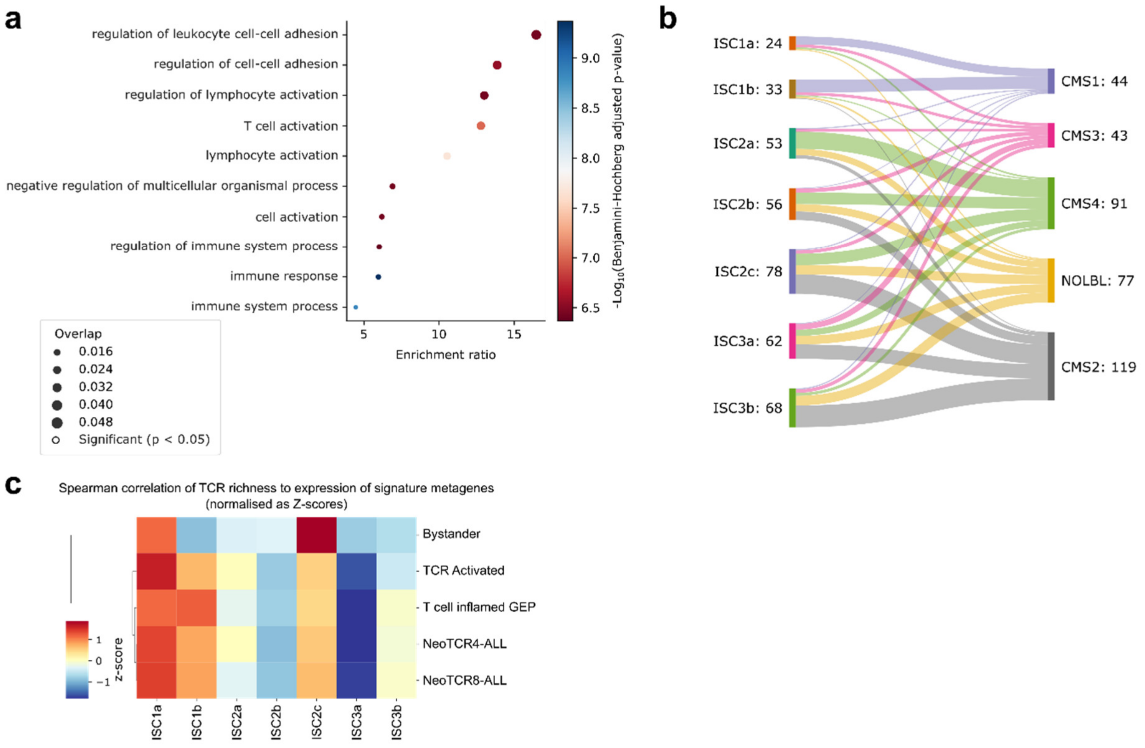

Supplementary Figure 6. Information transfer from single-cell transcriptomics to bulk RNA-seq in 374 TCGA patients with primary CRC tumours. (a) Network expansion analysis of tumour-reactive signalling genes in WebGestalt indicating the top 10 enriched categories sorted by enrichment ratio. Only hits with FDR-adjusted p-value < 0.05 and dot size by their enrichment ratio are shown. **(b)** Sankey plot highlighting how patients classify between the ISC and CMS system. The number of patients is shown as vertical bars per classification and the sizes of the strands represent the number of patients shared between each classification system. **(c)** Z-score normalised Spearman correlations between TCR richness and several gene expression signature metagenes for each ISC category. Correlations between TCR richness from Thorsson et al. (2018, Immunity) and the TCR activation gene signature, the bystander gene signature, the two NeoTCR gene signatures by Lowery et al. (2022, Science) and a T cell inflammation gene expression signature by Ayers et al. (2017, J Clin Invest) are shown.

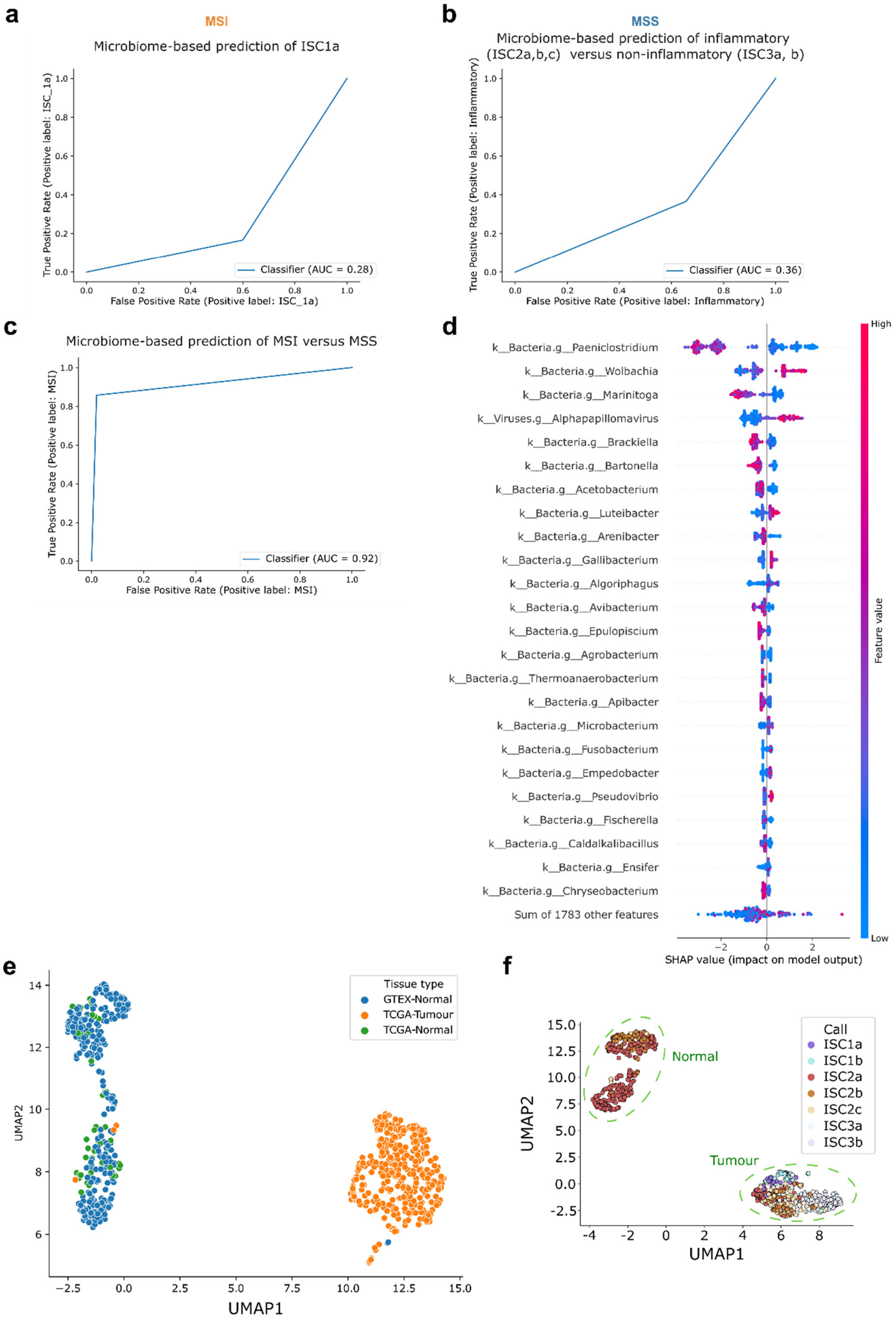

a
MSI
Microbiome-based prediction of ISC1a
True Positive Rate (Positive label: ISC_1a)
False Positive Rate (Positive label: ISC_1a)
Classifier (AUC = 0.28)
b
MSS
Microbiome-based prediction of inflammatory (ISC2a,b,c) versus non-inflammatory (ISC3a, b)
True Positive Rate (Positive label: Inflammatory)
False Positive Rate (Positive label: Inflammatory)
Classifier (AUC = 0.36)
c
Microbiome-based prediction of MSI versus MSS
True Positive Rate (Positive label: MSI)
False Positive Rate (Positive label: MSI)
Classifier (AUC = 0.92)
d
k__Bacteria.g__Paeniclostridium
k__Bacteria.g__Wolbachia
k__Bacteria.g__Marinitoga
k__Viruses.g__Alphapapillomavirus
k__Bacteria.g__Brackiella
k__Bacteria.g__Bartonella
k__Bacteria.g__Acetobacterium
k__Bacteria.g__Luteibacter
k__Bacteria.g__Arenibacter
k__Bacteria.g__Gallibacterium
k__Bacteria.g__Algoriphagus
k__Bacteria.g__Avibacterium
k__Bacteria.g__Epulopiscium
k__Bacteria.g__Agrobacterium
k__Bacteria.g__Thermoanaerobacterium
k__Bacteria.g__Apibacter
k__Bacteria.g__Microbacterium
k__Bacteria.g__Fusobacterium
k__Bacteria.g__Empedobacter
k__Bacteria.g__Pseudovibrio
k__Bacteria.g__Fischerella
k__Bacteria.g__Caldalkalibacillus
k__Bacteria.g__Ensifer
k__Bacteria.g__Chryseobacterium
Sum of 1783 other features
SHAP value (impact on model output)
High
Feature value
Low
e
Tissue type
GTEX-Normal
TCGA-Tumour
TCGA-Normal
UMAP2
UMAP1
f
Call
ISC1a
ISC1b
ISC2a
ISC2b
ISC2c
ISC3a
ISC3b
Normal
Tumour
UMAP2
UMAP1

Supplementary Figure 7. Random forests using the TCGA microbiome. Microbial data was downloaded from the Poore et al. (2020, Nature) study, in which reads from TCGA were aligned to bacterial and viral genomes with KRAKEN. Non-normalised reads post quality control filtering were used for the random forest classifiers. **(a)** ROC curve for the prediction of ISC1a in MSI (57 patients), using the microbiome reads. **(b)** ROC curve for the prediction of inflammatory ISC (ISC2a, b, c) in the TCGA MSS cohort (317 patients) using microbiome reads. **(c)** ROC curve for the prediction of microsatellite instability state (MSI as positive label) using microbial reads, using the 374 patient TCGA primary CRC cohort. **(d)** Top 25 most informative bacterial or viral genera as identified by the MSI versus MSS classifier. Shapley's feature importance metric is shown. **(e)** UMAP representing the different datasets (TCGA, GTex) and tissue states (normal, tumour) after Combat batch correction. Batch-corrected data were used as input for a random forest classifier used to call ISC subtypes in GTex and TCGA for these 363 normal tissue samples. All genes were used for the manifold projection. **(f)** UMAP representation illustrating distribution of ISC calls on samples from TCGA and GTex (TCGA primary: 374, TCGA normal adjacent: 56, GTex normal: 307). Dataset batch effects were minimised using Combat, inferring the transformation matrix using only normal tissue samples in TCGA and GTex and then applying this to all samples. Only genes used by the classifier were used for the manifold projection.

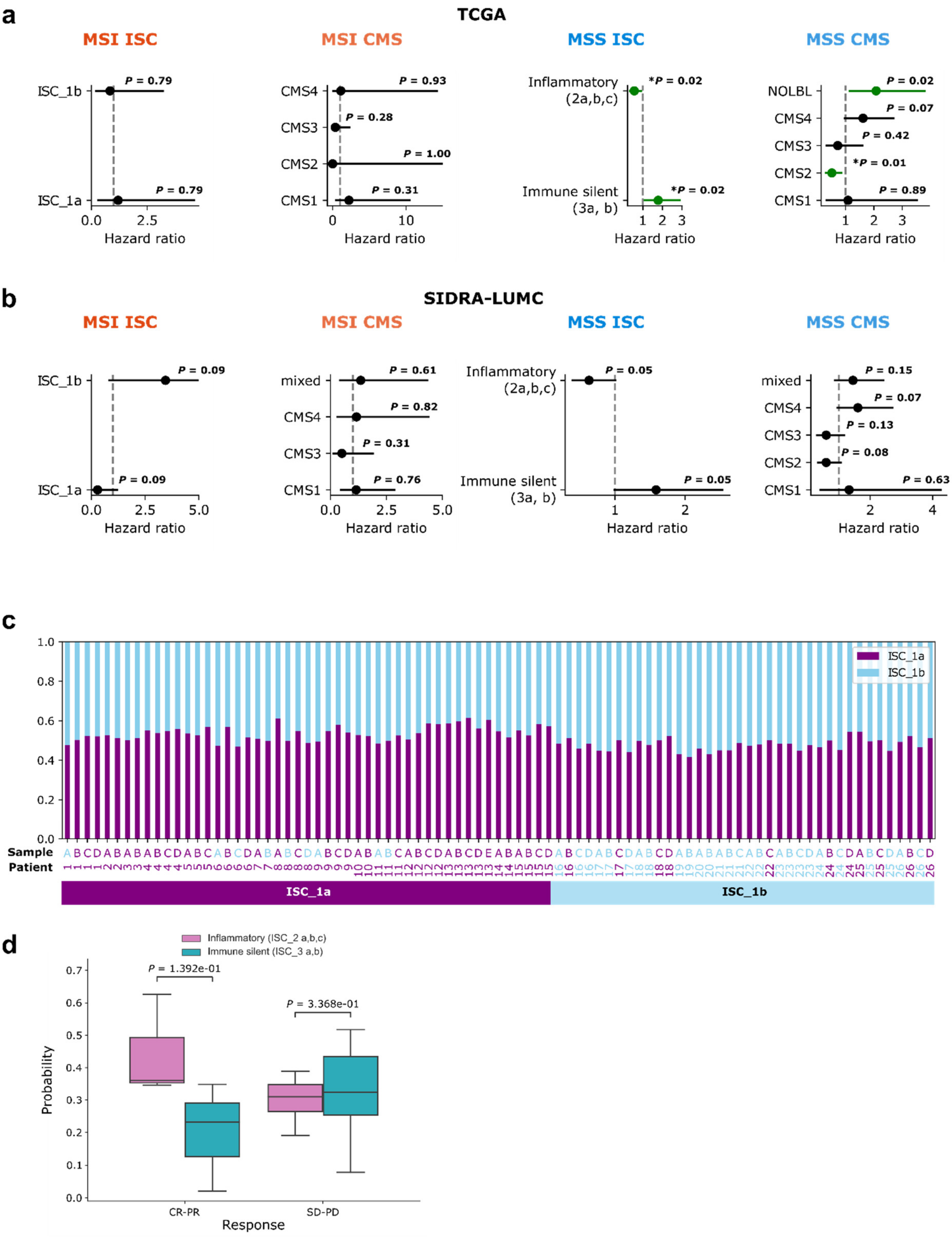
a
TCGA
MSI ISC
MSI CMS
MSS ISC
MSS CMS
ISC_1b
ISC_1a
P = 0.79
P = 0.79
CMS4
CMS3
CMS2
CMS1
P = 0.93
P = 0.28
P = 1.00
P = 0.31
Inflammatory (2a,b,c)
Immune silent (3a, b)
*P = 0.02
*P = 0.02
NOLBL
P = 0.02
P = 0.07
P = 0.42
*P = 0.01
P = 0.89
Hazard ratio
b
SIDRA-LUMC
MSI ISC
MSI CMS
MSS ISC
MSS CMS
P = 0.09
P = 0.09
mixed
P = 0.61
P = 0.82
P = 0.31
P = 0.76
P = 0.05
P = 0.05
P = 0.15
P = 0.07
P = 0.13
P = 0.08
P = 0.63
Hazard ratio
c
ISC_1a
ISC_1b
Sample
Patient
d
Inflammatory (ISC_2 a,b,c)
Immune silent (ISC_3 a,b)
P = 1.392e-01
P = 3.368e-01
Probability
CR-PR
SD-PD
Response

Supplementary Figure 8. Prognostic impact, tumoural heterogeneity and response indication in public patient cohorts. (a-b) Estimated hazard ratios of multivariate CoxPH regression model correcting for age, gender and tumour purity (TCGA, 374 patients, 52 MSI and 268 MSS with PFI, age, gender and purity annotated; **a**) or age and gender (SIDRA-LUMC, 348 patients, 57 MSI and 224 MSS with PFS, age and gender annotated ; **b**) when comparing the respective ISC or CMS ingroups to their outgroups (e.g. CMS1 vs not in CMS1). **(c)** Prediction of the ISC labels per macro-dissected region in the Keynote-177 MSI patient trial (26 patients available, unique samples indicated with a letter), suggesting that different tumoural regions may contain distinct immune landscapes covered by ISC. **(d)** Boxplot indicating the ISC class label probabilities for responder (CR/PR: 1 patient) and non-responders (SD/PD: 11 patients) patients in a MSS patient trial combining chemotherapy and radiotherapy (Parikh et al. (2021) Nat Cancer). All 32 publicly available samples, regardless of treatment state and patient were treated independently for statistics. Statistical significance was assessed with Welch's t-test. More data is required before any generalisable statements can be made for MSS CRC.
