# Supplementary material for: Single cell dynamics of tumor specificity vs bystander activity in CD8+ T cells define the diverse immune landscapes in colorectal cancer
Source: Cell Discov. 2023 Nov 15;9:114. doi: 10.1038/s41421-023-00605-4 (PMC10652011; doi:10.1038/s41421-023-00605-4)
Supplement: Supplementary file 4 — Supplementary Table S3 [file 41421_2023_605_MOESM4_ESM.pdf]

MAIT signature:
SLC4A10 ME1 COLQ NCR3 TLE1 ZBTB16 RORC KLRB1 CXXC5 PZP LST1 ABCB1 FSD1 P2RY14 A2M MYBL1 PLXND1 IFNGR1 SYTL2 IL18RAP TMIGD2 CEBPD DPP4 PRR5 IL23R FKBP11

CD8$^+$ T cell annotation signatures:
General CD8: *CD8A CD8B*
Subpopulation annotation: *B3GAT1 BACH2 BTLA CCL3 CCL4 CCL5 CCR7 CD244 CD27 CD28 CD44 CTLA4 CX3CR1 ENTPD1 EOMES GZMA GZMB GZMK HAVCR2 ICOS IDO1 IFNG IL12A IL13 IL17A IL17RA IL2 IL22 IL2RA IL2RB IL3 IL4 IL5 IL6 IL7R IL9 ITGAE JAK3 KLF2 KLRG1 LAG3 PDCD1 PRF1 PTPRC SELL SELP SPN STAT1 TBX21 TIGIT TNF TNFRSF4 TNFRSF8 TNFRSF9 TOX LTA*

MAIT TCR activation signatures:
Ribo$^+$ (TCR-dependent): *IL17A TNF FURIN HMGB1 HMGB2*
Ribo$^-$ (TCR-independent): *ZBTB16 GZMB PRF1 FASLG*
Activation: *IFNG IL2RA*
Tissue Residency: *CD69*
Inflammation: *TGFB1 NFKB1*

Effector signaling signature (correlation heatmap):
IFNG TNF CTLA4 HAVCR2 PDCD1 ENTPD1 TCF7 TOX EOMES TBX21 STAT1 STAT2 NFATC3 NFATC1 NFATC2 YY1 SMAD2 SMAD3 SMAD4 TIGIT LAG3 MAF NFIL3 PRDM1 IL2 CD28 JAK3 BCL6 SLAMF6 CXCR5 ICOS IL15RA IL18R1 IL18RAP KLRK1 KLRG1
CXCL13 CXCL9 CXCL10

Tumour reactive signalling modules:
MSI stem-like progenitor: *CD28 MAF TCF7 IL2*
MSI inflamed follicular-like: *YY1 NFATC1 BCL6 CXCL10 CXCR5 ICOS*
MSI long-term immunosurveillance: *PRDM1 TNF EOMES KLRG1 KLRK1 SLAMF6 TBX21 SMAD2 SMAD4 IL18R1*
MSI tumour-reactive exhaustion: *IFNG LAG3 JAK3 NFATC2 PDCD1 CTLA4 TIGIT ENTPD1 HAVCR2 CXCL13*
MSI memory differentiation: *STAT1 IL18RAP NFIL3 CXCL9 STAT2 SMAD3 IL15RA TOX NFATC3*
MSS long-term immunosurveillance: *IL2 IL18R1 KLRK1 TNF KLRG1 PRDM1 EOMES SLAMF6*
MSS inflamed follicular-like: *CD28 BCL6 CXCR5*
MSS Inflamed memory-like: *NFATC2 CTLA4 PDCD1 TOX NFATC3 SMAD2 IL18RAP IL15RA SMAD3 SMAD4 STAT2 NFATC1 STAT1 JAK3*
IFNγ-associated dysfunctional: *YY1 TBX21 NFIL3 CXCL9 CXCL10*
Tolerogenic c-Maf signalling: *TCF7 MAF ICOS LAG3 TIGIT ENTPD1 HAVCR2 CXCL13*

NeoTCR4-All signature:
Lowery et al. (2022) Science: https://doi.org/10.1126/science.abl5447
CXCL13 HMOX1 ETV7 ADGRG1 PDCD1 ENTPD1 CCDC50 TOX CD4 TIGIT TNFRSF18 NMB MYL6B AHI1 MAF IFNG LAG3 CXCR6 IGFLR1 DUSP4 ACP5 LINC01943 LIMS1 BATF PCED1B ITGAL YPEL2 MAL PPT1 ELMO1 MIS18BP1 TMEM173 ADI1 SLA GALM LBH SECISBP2L CTSB C17orf49 CORO1B CARHSP1 SRPK2 ARL3 PTMS CD82 HNRNPLL CTSC LINC01871 CCDC167 SMC3 PPM1G ORMDL3 VPS25 BST2 TRAF3IP3 NAP1L4 HLA-DPA1 PIM2 SH2D1A RILPL2 CCNDBP1

NeoTCR8-All signature:
Lowery et al. (2022) Science: https://doi.org/10.1126/science.abl5447
ATP10D GZMB ENTPD1 KIR2DL4 LAYN HTRA1 CD70 CXCR6 HMOX1 ADGRG1 LRRN3 ACP5 CTSW GALNT2 LINC01480 CARS LAG3 TOX PTPRCAP ASB2 ITGB7 PTMS CD8A GPR68 NSMCE1 ABI3 SLC1A4 PLEKHF1 CD8B LINC01871 CCL4 NKG7 CLIC3 NDFIP2 PLPP1 PCED1B CXCL13 PDCD1 PRF1 HLA-DMA GPR25 CD9 TIGIT HLA-DRB5 SYTL3 SLF1 NEK1 CASP1 SMC4 TSEN54 PLSCR1 GNPTAB HLA-DPB1 PLEKHA1 ARHGAP9 ALOX5AP SH3BP1 NCF4 NELL2 GATA3 PPM1M TNFRSF1A AC022706.1 MCM5 HLA-DRB1 TNFSF10 TRIM21 HDLBP ERN1 CALHM2 SASH3 ACTA2 MAST4 CAPG MPST IGFLR1 GZMA CD27 ITGAE SLA2 RHOC COMMD8 MYO1G SP140 PHPT1 CD2BP2 PLEKHO1 STAM MRPL16 IL2RB ID2 TESPA1 GOLGA8B MIS18BP1 VAMP5 DAPK2 HLA-DPA1 TSG101 IL4R CCND2 CTSC TRAF3IP3 NLRC3 ORAI3 GNLY MIR155HG CARD16 CD82 ECH1 JAML EEF1G ETFB DAXX RBM4 HCST RAB27A YPEL2 CHST12 ARPC1B PDIA4 PDIA6 AC243960.1 TBC1D10C PTPN6 PYCARD BST2 BTN3A2 MTG1 MLEC DUSP4 GSDMD SLAMF1 IFI6 PCID2 GIMAP1 ITGA1 CSNK2B CDK2AP2 MYO1F AC004687.1 PTTG1 APOBEC3C TSPAN14 MOB3A STXBP2 LCP2 PLA2G16 LINC00649 CST7 TADA3 SIT1 APOBEC3G SUSD3 CD3G CCL5 CDC25B TNFRSF1B HMGN3 THEMIS ASF1A CTNNB1 FIBP CCDC85B POLR3GL GIMAP6 ARL6IP1 CALCOCO2 CCPG1 KLRB1 ACAA2 ISG15 EIF4A1 CAT MANF XAB2 GRINA GLO1 LSM2 SLFN5 FKBP1A AKNA TAP1 LMO4 APEH C12orf75 TMEM14A DNPH1 C17orf49 NUDT5 MGAT1 CCDC69 EIF4EBP1 PDHB ARL3 UCP2 IFI35 HSBP1 LYST MRFAP1L1 ITGAL AIP RASAL3 CAPN1 ITGB1 RBPJ LBH DYNLL1 NME2 MT1F SYNGR2 ABTB1 ZGPAT CD63 ILK SKA2 TMEM204 ACO2 HOPX CRIP1 OXNAD1 CCS GRAP2 GSTO1 HADHB IL16 PIN4 CUEDC2 CALM3 SAMSN1 HM13 SNAP23 LPCAT4 FAAP20 EFHD2 PRDX3 CCM2 C22orf39 SDHA ARRDC1 MAP4K1 NDUFA13 IL27RA C14orf119

Bystander activation signature:
Bangs et al. (2009) J Immunol: https://doi.org/10.4049/jimmunol.0802596
ADAM8 ADGRE5 AK2 ALOX5 ANTXR2 ANXA2 ANXA2P2 ARHGAP21 ATG2A ATP1B1 ATP1B3 ATP5MK BATF BCAT2 BCL2L1 BHLHE40 BIRC3 BYSL C12orf75 C4orf46 CDK5 CENPJ CHST11 CLDND1 CLIC1 COPS8 COX5A CSF2RB CSTF2 CTSH CTTNBP2NL CYFIP1 DBI DESI2 DHRS7B DLST DMD DPP4 DUSP2 DYNLL1 DYNLT2B EBP EGR3 EIF3J EIF4E2 ELOC ELOVL1 ELOVL5 ENO1 ERP44 ETFDH FAHD2A FOSL2 FRMD4B FUCA2 GCA GK GNA15 GNG2 GPR68 GRAMD4 HBEGF HCST HDAC9 HEATR6 HK1 HLF HNRNPLL IDH1 IKBIP IKZF2 IKZF4 IL12RB2 IL17RB IL2RB IRAK2 IRF4 ITPRIPL1 KAT2B KIF3B KLHL2 LAMTOR2 LAYN LDLRAD4 LGALS1 LINC00888 LPP LRP8 LRRC59 LTA LYRM1 MAF MAP2K3 MAP3K8 MAST4 MBD2 MCTS1 METTL7A MGST2 MIR155HG MLX MRPL42 MRPL47 MTHFD1 MTHFD1L MYB MYCBP MYL12A MYO5A NAMPT NCF4 NDUFA6 NDUFB8 NEDD9 NFKBIE NOD2 NSD2 OSM PANX1 PDE4A PDE4B PDHA1 PEA15 PHTF2 PIGX PKD2 PLAGL2 PLPP1 PMCH PNKP PPP1R16B PPP1R18 PRDM1 PRMT9 PTPN22 PTTG1 RAB11FIP1 RAC2 RALB RANBP9 RBBP8 RCBTB2 RFTN1 RGS19 RILPL2 RTKN2 RUNX3 SAP30 SAT1 SC5D SEC23B SEC61B SELENOS SGK1 SH3BP5 SHMT2 SIPA1L1 SLA2 SLAMF1 SLC39A14 SLCO4A1 SMAD7 SMIM30 SNAP47 SNRPF SNX10 SOCS2 SRP19 SRPK1 STAM STING1 STK24 SWAP70 TAGLN2 TBC1D2 TBCE TESC TFRC TGFBR1 TIPIN TJP2 TM9SF4 TMEM167A TNF TNFRSF18 TNFRSF1B TNFRSF9 TNFSF12 TOR3A TRIB1 TTC9C TXNL4A UEVLD UHRF1BP1L UTP11 WIPI1 YIPF6 ZBTB8OS ITPRIPL1 KAT2B KIF3B KLHL2 LAMTOR2 LAYN LDLRAD4 LGALS1 LINC00888 LPP LRP8 LRRC59 LTA LYRM1 MAF MAP2K3 MAP3K8 MAST4 MBD2 MCTS1 METTL7A MGST2 MIR155HG MLX MRPL42 MRPL47 MTHFD1 MTHFD1L MYB MYCBP MYL12A MYO5A NAMPT NCF4 NDUFA6 NDUFB8 NEDD9 NFKBIE NOD2 NSD2 OSM PANX1 PDE4A PDE4B PDHA1

TCR activation signature:
Bangs et al. (2009) J Immunol: https://doi.org/10.4049/jimmunol.0802596
ADAT2 AGPAT5 ANTXR2 ARNTL2 ATP5MJ B3GNT5 B3GNTL1 BCAT1 BCL2L1 BMP2K BOLA3 BPNT2 BTBD3 BYSL C19orf54 CACNA2D4 CAMK2D CCDC141 CCT8 CD109 CD72 CHMP5 CHP1 CIAO2B CLYBL CMTM7 COMMD3 COPRS COPS8 COX5A CPPED1 CREB3L2 DBI DELE1 DHCR24 DHRSX DMAC1 DPP4 DUSP2 EFHD2 EGR2 EIF2B2 EIF2S2 EIF3J EMILIN2 EPAS1 ERP44 EVI5 F5 FAH FAM210A FAM221A FDFT1 FDX1 FGGY FOSL2

GCA GEM GK GM2A GNA12 GNA15 GNG5 GNGT2 GNPAT GPRIN3 GRAMD1B GRAMD4 GSDME GTDC1 GTF2H5 HDLBP HIVEP1 HS2ST1 HSD17B10 IER3 INPP1 IRAK1BP1 IRAK2 IRF4 ITGAE ITGAV ITGAX KCNN4 KYAT3 LAG3 LANCL2 LDLRAD4 LPP LRRC28 LRRCC1 LYPLA1 LYRM4 MBD2 MCTP2 MEAK7 METRNL MIR155HG MRPL42 MRPL47 MSMO1 MTMR2 NDUFA7 NDUFA8 NDUFAB1 NDUFB5 NEDD9 NHS NME7 NOD2 NR4A3 PAQR6 PARK7 PDE4D PFDN4 PGAM1 PIGX PNKP PPP1R16B PPP2R5A PRDM1 PRPF6 PTTG1 QSOX2 RAB13 RAB37 RAC2 RASSF2 RCAN2 RDH10 RDX RFTN1 RGL4 RNF7 SARNP SCP2 SEC11A SEC61A2 SEC61G SELENOS SFT2D1 SIRPG SLC12A6 SLC15A4 SLC25A13 SLC25A17 SLC31A1 SLC35G2 SLC39A14 SLC7A5 SLC9B2 SLCO4A1 SLIT1 SNRPF SOCS6 SPACA9 SPATS2L SPRY1 SPTSSA SQLE SS18 ST8SIA4 STAG3 STAM STAMBP STYXL1 SUOX SYTL3 TBCE TFRC THOC7 TIMM23 TJP2 TM9SF4 TMEM185A TNFRSF11A TNFRSF1B TNFRSF9 TNIP3 TPM4 TRAK1 TTN TXNDC17 TXNL4A UBE2G1 UEVLD UHRF1BP1L URM1 VPS8 VSIG10L WDR12 WIPI1 WSB2 YARS1 ZADH2 ZFYVE1 ZMIZ1 ZNF282 ZNF57 ZNRF1

IFNg score:

Ayers et al. (2017) J Clin Invest : https://doi.org/10.1172/JCI91190

IDO1 CXCL10 CXCL9 HLA-DRA STAT1 IFNG

T cell inflamed GEP:

Ayers et al. (2017) J Clin Invest : https://doi.org/10.1172/JCI91190

TIGIT CD27 CD8A PDCD1LG2 LAG3 CD274 CXCR6 CMKLR1 NKG7 CCL5 PSMB10 IDO1 CXCL9 HLA-DQA1 CD276 STAT1 HLA-DRB1 HLA-E

Sana IFNg response:

Sana et al. (2005) Cytokine : https://doi.org/10.1016/j.cyto.2004.11.003

APOL1 APOL2 APOL3 APOL4 ATP6V0A4 BATF2 BST2 C1S CASP1 CD274 CD74 CEACAM1 CFH CX3CL1 CXCL10 CXCL11 CXCL9 DDX60 DTX3L ETV7 GBP1 GBP3 GBP4 GIMAP7 GOLM1 HLA-A HLA-B HLA-C HLA-DMA HLA-DPA1 HLA-DQA1 HLA-DQB1 HLA-DRB1 HLA-DRB3 HLA-DRB4 HLA-DRB5 HLA-E HSD17B11 IDO1 IFI35 IFI44L IFIH1 IL18BP IL23A LAP3 LGALS3BP LGALS9 LIPG MLKL MMP25 MX1 NLRC5 OAS1 OAS2 PARP14 PARP9 PLA1A PLAAT4 PPP3CA RABL3 RAC3 RNF213 SAMD9L SAMHD1 SEPTIN4 SERPING1 SLC25A28 SSPN ST8SIA4 STAT1 TNFSF10 TRIM22 UBD UBE2L6 VAMP5 WARS1
