# Supplementary material for: Single cell dynamics of tumor specificity vs bystander activity in CD8+ T cells define the diverse immune landscapes in colorectal cancer
Source: Cell Discov. 2023 Nov 15;9:114. doi: 10.1038/s41421-023-00605-4 (PMC10652011; doi:10.1038/s41421-023-00605-4)
Supplement: Supplementary file 5 — Supplementary Table S4 [file 41421_2023_605_MOESM5_ESM.pdf]

Explanation of literature-based nomenclature of the distinct signalling modules in MSI or MSS CRC *IFNG*$^+$CD8$^+$ T cells

- **MSI CRC *IFNG*$^+$CD8$^+$T cells**
  - **Stem-like progenitor:** This cluster had a distinct co-expression of *TCF7, IL2,* and *CD28*. It has been reported that stem-like precursor CD8$^+$ T cells highly express TCF1 (coded by *TCF7*) together with some effector markers like CD28, further complemented by self-renewal capacity via pro-proliferation cytokines like IL2 [1]. Hence, we termed this cluster as 'stem-like progenitor' signalling module.
  - **Inflamed follicular-like:** This cluster had a distinct co-expression of *CXCR5, BCL6,* and *ICOS*. Herein, BCL6 and CXCR5 are well-established markers of follicular-like T cell phenotype wherein co-presence of ICOS marks a critical inflammatory signal to further facilitate BCL6/CXCR5 associated follicular T cell differentiation [2]. Hence, we termed this cluster as 'inflamed follicular-like' signalling module.
  - **Long-term immunosurveillance:** This cluster had a co-expression of *EOMES, TBX21, PRDM1, KLRK1,* and *KLRG1*. It has been reported that *EOMES*, *TBX21* and *PRDM1* together form the signalling 'core' of long-lived CD8$^+$ T cells that perform MHC-dependent immunosurveillance [3,4]. Similarly, *KLRK1* and *KLRG1* are well-established memory-like phenotype markers that also associate with temporally sustained immunosurveillance [3,4]. Given all the literature evidence, we named this group 'long-term immunosurveillance'.
  - **Tumour-reactive exhaustion:** This cluster had a co-expression of *IFNG, LAG3, PDCD1, CTLA4, TIGIT, ENTPD1, HAVCR2, CXCL13, NFATC2*. It has long been established that co-enrichment of effector genes like *IFNG*, *NFATC2*, *CXCL13*, together with immune-inhibitory receptors like LAG3, PDCD1, CTLA4, TIGIT, *ENTPD1* and *HAVCR2*/TIM3 marks CD8$^+$ T cells that are tumour-reactive and show exhaustion on account of overexposure to tumoural antigens [5–9]. Hence, taken together, we termed this cluster as 'tumour-reactive exhaustion' signalling module.
  - **Memory differentiation:** In this cluster, we identified co-expression of *TOX, IL15RA, NFATC3, NFIL3,* and *STAT1/2*. Genes like *TOX*, *NFATC3* and *NFIL3* mark memory differentiation in T cells such that effector programs linked to *IL15RA* and *STAT1/2* further potentiate or accompany pro-memory phenotypes [1,10]. Hence, 'memory differentiation' appeared to be the most accurate description for this cluster.

- **MSS CRC *IFNG*⁺CD8⁺T cells**
  - **Long-term immunosurveillance:** This cluster had a co-expression of *EOMES, PRDM1, KLRK1, KLRG1, IL2,* and *TNF*. As mentioned above, at least *EOMES* and *PRDM1* are within the signalling 'core' of long-lived CD8⁺T cells performing MHC-dependent immunosurveillance [3,4]. Similarly, *KLRK1* and *KLRG1* co-mark memory-like phenotype also associated with temporally sustained immunosurveillance [3,4]. Finally, presence of IL2 and TNF marks an effector T cell phenotype, which is functionally like the effector transcriptional programs sustained by *TBX21* in T cells [3,4]. As such, 'long-term immunosurveillance' appeared to be the most appropriate description of this signalling module.
  - **Inflamed follicular-like:** This cluster had a distinct co-expression of *CXCR5, BCL6,* and *CD28*. As mentioned above, BCL6 and CXCR5 are well-established markers of follicular-like T cell phenotype [2]. Herein, ICOS and CD28 are homologues of each other [2], so while unlike MSI CRC above, this cluster did not have ICOS co-expression yet presence of CD28 can still mark increased inflammatory differentiation of follicular T cells [11]. Therefore, we termed this cluster as 'inflamed follicular-like'.
  - **Inflamed memory-like:** This cluster had a co-expression of *TOX, PDCD1, CTLA4, NFATC2/3, SMAD2/3/4,* and *STAT1/2, IL15RA, IL18RAP*. *TOX* and *NFATC2/3* mark memory differentiation in T cells such that effector programs linked to *IL15RA* and *STAT1/2* further potentiate or accompany pro-memory phenotypes [1,10]. Beyond this, this cluster also included a mixture of immune-inhibitory receptors (*PDCD1*, *CTLA4*), cytokine signalling (*IL18RAP*) and immuno-regulatory signalling (*SMAD2/3/4*) [12–14]. These ambiguously directed pathway together mark general inflammation, hence the label 'inflamed memory-like' for this signalling module.
  - **IFNγ-associated dysfunctional:** This cluster had co-expression of IFN-γ-related genes like *TBX21, YY1, NFIL3, CXCL9, CXCL10* but negative correlations with other functional genes like *IL2, TNF, KLRK1*. Such contradictions are signs of dysfunction originating from incomplete or inappropriate activation of T cells, because correctly activated T cells tightly co-express activation programs associated with IFNG, IL2 and TNF [15–18]. We therefore describe this cluster as 'IFNγ-associated dysfunctional'.
  - **Tolerogenic c-Maf signalling:** This cluster had a co-expression of *MAF, ICOS, LAG3, TIGIT, ENTPD1, and HAVCR2*. It has been reported that ICOS is one of the inducers of MAF expression whereas *LAG3*, *TIGIT*, *ENTPD1* and *HAVCR2* are different immune-inhibitory receptor coding genes that are induced by the MAF-driven program [19]. Due to the omnipresence of upstream and downstream signalling centred around MAF, which tends to

have highly tolerogenic activity at colonic surfaces [19], we defined this cluster as the 'tolerogenic c-Maf signalling' signalling module.

REFERENCES:

1. Escobar G, Mangani D, Anderson AC. T cell factor 1: A master regulator of the T cell response in disease. *Sci Immunol* 2020; **5**.

2. Choi YS, Kageyama R, Eto D, *et al.* ICOS receptor instructs T follicular helper cell versus effector cell differentiation via induction of the transcriptional repressor Bcl6. *Immunity* 2011; **34**:932–946.

3. McMurray JL, von Borstel A, Taher TE, *et al.* Transcriptional profiling of human Vδ1 T cells reveals a pathogen-driven adaptive differentiation program. *Cell Rep* 2022; **39**:110858.

4. Pauken KE, Shahid O, Lagattuta KA, *et al.* Single-cell analyses identify circulating anti-tumor CD8 T cells and markers for their enrichment. *J Exp Med* 2021; **218**.

5. Li H, van der Leun AM, Yofe I, *et al.* Dysfunctional CD8 T Cells Form a Proliferative, Dynamically Regulated Compartment within Human Melanoma. *Cell* 2019; **176**:775-789.e18.

6. McLane LM, Abdel-Hakeem MS, Wherry EJ. CD8 T cell exhaustion during chronic viral infection and cancer. *Annu Rev Immunol* 2019; **37**:457–495.

7. Chow A, Perica K, Klebanoff CA, Wolchok JD. Clinical implications of T cell exhaustion for cancer immunotherapy. *Nat Rev Clin Oncol* 2022; **19**:775–790.

8. Naulaerts S, Datsi A, Borras DM, *et al.* Multiomics and spatial mapping characterizes human CD8+ T cell states in cancer. *Sci Transl Med* 2023; **15**:eadd1016.

9. Vanmeerbeek I, Borras DM, Sprooten J, Bechter O, Tejpar S, Garg AD. Early memory differentiation and cell death resistance in T cells predicts melanoma response to sequential anti-CTLA4 and anti-PD1 immunotherapy. *Genes Immun* 2021; **22**:108–119.

10. Curdy N, Lanvin O, Laurent C, Fournié J-J, Franchini D-M. Regulatory mechanisms of inhibitory immune checkpoint receptors expression. *Trends Cell Biol* 2019; **29**:777–790.

11. Wang CJ, Heuts F, Ovcinnikovs V, *et al.* CTLA-4 controls follicular helper T-cell differentiation by regulating the strength of CD28 engagement. *Proc Natl Acad Sci USA* 2015; **112**:524–529.

12. Hackstein C-P, Spitzer J, Symeonidis K, *et al.* Interferon-induced IL-10 drives systemic T-cell dysfunction during chronic liver injury. *J Hepatol* 2023; **79**:150–166.

13. Hedl M, Zheng S, Abraham C. The IL18RAP region disease polymorphism decreases IL-18RAP/IL-18R1/IL-1R1 expression and signaling through innate receptor-initiated pathways. *J Immunol* 2014; **192**:5924–5932.

14. Banas MC, Parks WT, Hudkins KL, *et al.* Localization of TGF-beta signaling intermediates Smad2, 3, 4, and 7 in developing and mature human and mouse kidney. *J Histochem Cytochem* 2007; **55**:275–285.

15. Flemming A. What's driving T cell dysfunction? *Nat Rev Cancer* 2019; **19**:251.

16. Salerno EP, Shea SM, Olson WC, *et al.* Activation, dysfunction and retention of T cells in vaccine sites after injection of incomplete Freund's adjuvant, with or without peptide. *Cancer Immunol Immunother* 2013; **62**:1149–1159.

17. Djuretic IM, Levanon D, Negreanu V, Groner Y, Rao A, Ansel KM. Transcription factors T-bet and Runx3 cooperate to activate Ifng and silence Il4 in T helper type 1 cells. *Nat Immunol* 2007; **8**:145–153.

18. Collins PL, Chang S, Henderson M, *et al.* Distal regions of the human IFNG locus direct cell type-specific expression. *J Immunol* 2010; **185**:1492–1501.

19. Imbratta C, Hussein H, Andris F, Verdeil G. c-MAF, a Swiss Army Knife for Tolerance in Lymphocytes. *Front Immunol* 2020; **11**:206.
